# Natural Products from Marine-Derived Fungi with Anti-Inflammatory Activity

**DOI:** 10.3390/md22100433

**Published:** 2024-09-25

**Authors:** Yikang Qiu, Shiji Chen, Miao Yu, Jueying Shi, Jiayu Liu, Xiaoyang Li, Jiaxing Chen, Xueping Sun, Guolei Huang, Caijuan Zheng

**Affiliations:** 1 Key Laboratory of Tropical Medicinal Resource Chemistry of Ministry of Education, College of Chemistry and Chemical Engineering, Hainan Normal University, Haikou 571158, China; qyk7747226@sina.com (Y.Q.); chenshijinpc@126.com (S.C.); yumiaonpc@126.com (M.Y.); jueying202406@163.com (J.S.); gfjyyqh7154@outlook.com (J.L.); 13683798967@163.com (X.L.); 18976052357@163.com (J.C.); 2 Key Laboratory of Tropical Medicinal Plant Chemistry of Hainan Province, Haikou 571158, China; 3 Key Laboratory of Common Technology of Traditional Chinese Medicine Preparation, College of Pharmacy, Guangxi University of Chinese Medicine, Nanning 530200, China

**Keywords:** marine-derived fungi, marine natural products, anti-inflammatory

## Abstract

Inflammation is considered as one of the most primary protective innate immunity responses, closely related to the body’s defense mechanism for responding to chemical, biological infections, or physical injuries. Furthermore, prolonged inflammation is undesirable, playing an important role in the development of various diseases, such as heart disease, diabetes, Alzheimer’s disease, atherosclerosis, rheumatoid arthritis, and even certain cancers. Marine-derived fungi represent promising sources of structurally novel bioactive natural products, and have been a focus of research for the development of anti-inflammatory drugs. This review covers secondary metabolites with anti-inflammatory activities from marine-derived fungi, over the period spanning August 2018 to July 2024. A total of 285 anti-inflammatory metabolites, including 156 novel compounds and 11 with novel skeleton structures, are described. Their structures are categorized into five categories: terpenoids, polyketides, nitrogen-containing compounds, steroids, and other classes. The biological targets, as well as the in vitro and in vivo screening models, were surveyed and statistically summarized. This paper aims to offer valuable insights to researchers in the exploration of natural products and the discovery of anti-inflammatory drugs.

## 1. Introduction

Inflammation is considered as one of the most prime protective innate immunity responses, which is derived from the process of activation caused by the mammalian immune system. Inflammation is closely related to the body’s defense mechanism, which responds to chemical, biological, or physical infections and damages [1,2]. Furthermore, chronic inflammation, characterized by uncontrolled inflammation, can evolve into a persistent issue. It significantly contributes to the development of various diseases, including asthma, diabetes, Alzheimer’s disease, atherosclerosis, rheumatoid arthritis, and even certain cancers. Consequently, managing chronic inflammation and maintaining a balance between inflammatory and anti-inflammatory processes within the body is of considerable importance [3,4,5].

The revival of normal tissue homeostasis after exposure to injurious conditions is a beneficial consequence of inflammation, primarily orchestrated by a complicated set of defensive feedback interactions between soluble inflammatory mediators and cells [6,7,8]. In the course of inflammation, activated immune monocytes and macrophages initiate the transactivation of various critical transcription factors. The well-known inflammatory signal pathway, the NF-*κ*B (nuclear factor kappa-B) signal pathway, is classified as a canonical pathway. The activation of NF-*κ*B enhances the activity of downstream responses, including the production of pro-inflammatory cytokines such as interleukin-1*β* (IL-1*β*), interleukin-6 (IL-6), tumor necrosis factor (TNF-*α*), etc. It also increases the activity of pro-inflammatory enzymes, like inducible nitric oxide synthase (iNOS) and cyclooxygenase 2 (COX-2), among others, leading to the generation of nitric oxide (NO) and prostaglandin E2 (PGE2) [9,10]. The mitogen-activated protein kinase (MAPK) signaling pathway, which includes extracellular signal-regulated kinases (ERK), p38 MAPK, and c-Jun NH2-terminal kinases (JNK), can also be activated by inflammation. This pathway regulates the transcription of various genes associated with inflammation, thereby amplifying the downstream inflammatory response. A multitude of inflammatory mediators and factors contribute to cellular damage and inflammation, manifesting as symptoms such as redness, pain, fever, and swelling [11,12]. Consequently, targeting the reduction in these pro-inflammatory mediators can be an effective strategy for controlling and preventing chronic inflammatory diseases to a certain degree. Researchers typically assess anti-inflammatory activity by monitoring the suppressed expression of pro-inflammatory cytokines, the pro-inflammatory enzyme of COX-2, iNOS, and their derived production. A variety of proteins associated with inflammation were also tested in NF*-κ*B and MAPK signal pathways. These results were obtained from immune monocytes and macrophages, including BV2 and RAW264.7 cell lines, both in vitro, upon stimulation with lipopolysaccharides (LPS), and in vivo, by assessing the reduced swelling rate in a mouse ear edema model induced by phorbol myristate acetate (PMA) [13,14,15].

Toward the aim of discovering new natural products with anti-inflammatory activities, researchers discover novel sources in different environments. The oceans, with their unique aquatic environment and plentiful biodiversity, have garnered significant interest due to their rich reservoir of diverse secondary metabolites exhibiting potent anti-inflammatory, antitumor, antimicrobial, antiviral, antimalarial, and anti-oxidant activities. Marine natural products are of great value in drug development; approximately 20 natural lead molecules or compounds isolated from marine organisms have already become drugs, and even more are in clinical trials and the drug discovery pipeline [16,17,18,19]. For example, lurbinectedin, isolated from *Ecteinascidia turbinata* (tunicate), was granted orphan status and approved for the treatment of adult metastatic small-cell lung cancer (SCLC) in 2020 by the US FDA. It can reduce tumor-associated macrophages and the inflammatory tumor microenvironment in preclinical models [20]. Echinochrome A, a natural polyhydroxy-1,4-naphthoquinone pigment from the sea urchin *Scaphechinus mirabilis*, has been in clinical use since 1999. The drug exerts a therapeutic cytoprotective effect and is predominantly utilized in Russia to treat a variety of diseases, including the degeneration of the macula, retina, and cornea, circulatory disorder of the retina, and myocardial ischemia/reperfusion injury. Moreover, echinochrome A effectively normalizes lipid metabolism, recovers antioxidant status, and reduces atherosclerotic inflammation. It serves as both an anti-inflammatory and as an anti-oxidant agent and is also advantageous for the treatment of atherosclerosis and metabolic-syndrome-related diseases [21].

Among marine organisms, marine microorganisms are prolific producers of a multitude of novel bioactive secondary metabolites, which hold the potential to serve as promising lead molecules for drug development. Notably, marine-derived fungi play a vital role in the discovery of new anti-inflammatory drugs. Many novel secondary metabolites showing potent anti-inflammatory activities have been discovered from fungi that inhabit marine environments, including seawater, mangroves, sponges, corals, and sediments. Owing to their distinctive mechanisms of action, compounds derived from marine fungi have garnered increasing attention and have become one of the main areas of focus for the development of anti-inflammatory drugs [22,23].

Herein, 285 anti-inflammatory compounds reported from the beginning of August 2018 to July 2024 are included, and 96 references are cited in this review. This study comprehensively covers the chemical diversity and anti-inflammatory properties of 285 reported compounds, including 156 new compounds isolated from marine-derived fungi. The relevant biological and pharmacological activities of some potential compounds are also highlighted. Meanwhile, the structure–activity relationships of compounds with analogous chemical structures are discussed, which will benefit future drug development and innovation. This review provides valuable insights for researchers in the field of marine anti-inflammatory pharmacology and emphasizes the need for further research to harness the pharmacological benefits of marine-fungi-derived compounds for the development of effective and safe therapeutic agents.

## 2. Structural and Biological Activity Studies

Based on the literature search, 285 anti-inflammatory properties (**1**–**285**) were obtained from marine-derived fungi from 2018 to 2024. These compounds were structurally categorized into 53 terpenoids compounds, 110 polyketides compounds, 76 nitrogen-containing compounds, 20 steroid compounds, and 26 other compounds. The structures and the absolute configurations of the new and novel skeleton compounds were elucidated by a detailed spectroscopic analysis of NMR and MS data, time-dependent density functional theory (TDDFT)/ECD calculations, DP4+ probability predictions, single-crystal X-ray diffraction, and the Marfey, Snatzke, and Mosher methods.

### 2.1. Terpenoids

Terpenoids are a class of natural compounds derived from isoprene or isopentane. Structurally, they can be categorized into monoterpenoids, sesquiterpenoids, diterpenoids, sesterterpenes, triterpenoids, meroterpenoids, and polyterpenes. A total of 53 anti-inflammatory terpenoids were found from the marine-derived fungi, comprised of two monoterpenoids, 20 sesquiterpenoids, 21 diterpenoids, and 10 meroterpenoids. Among them, 44 were new compounds, and two new compounds (**1** and **46**) had novel skeleton structures.

#### 2.1.1. Monoterpenoids

**Figure 1:**
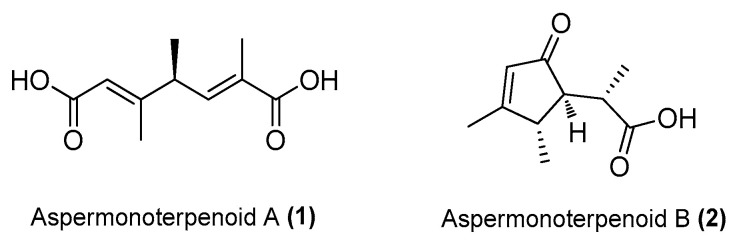
Anti-inflammatory monoterpenoids produced by marine fungi (**1**, **2**).

**Scheme 1:**
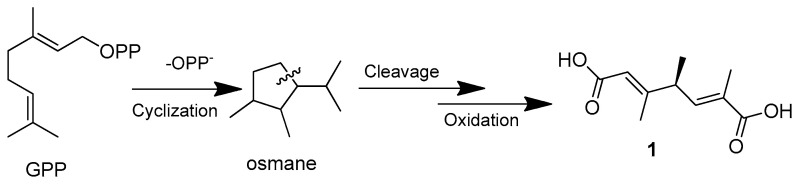
Plausible biogenetic pathway for compound **1** [24].

Two new osmane-related monoterpenoids, aspermonoterpenoids A (**1**) and B (**2**), were isolated from the deep-sea sediment-derived fungus, *Aspergillus sydowii* MCCC 3A00324 (South Atlantic Ocean) (Figure 1). Compound **1** possessed a novel monoterpenoid skeleton, and a plausible biosynthetic pathway for this compound was proposed in [24]. It is likely that **1** originated from the osmane-type monoterpenoid after cyclopentane ring cleavage and oxidation reactions (Scheme 1) [24]. Additionally, compounds **1** and **2** exhibited dose-dependent inhibitory activity against NO production in lipopolysaccharide (LPS)-induced RAW 264.7 cells at the concentrations of 20 and 10 µM, respectively [24].

#### 2.1.2. Sesquiterpenes

**Figure 2:**
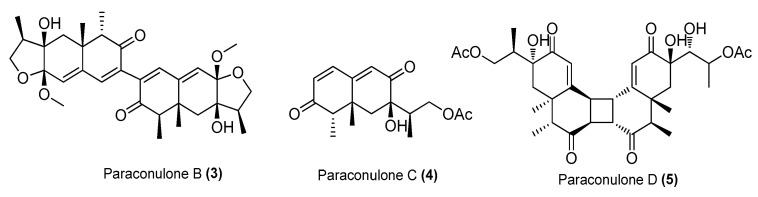

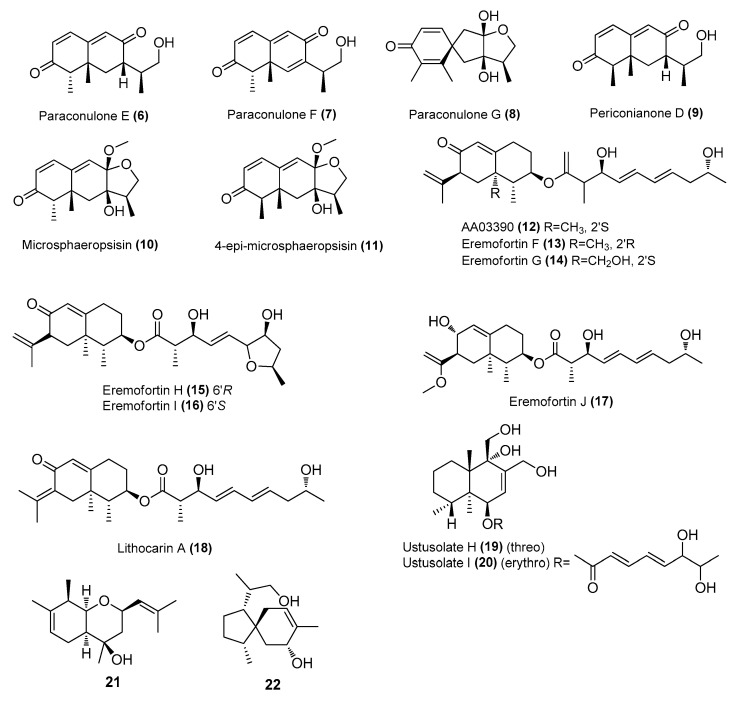
Anti-inflammatory sesquiterpenes produced by marine fungi (**3**–**22**).

Six new eremophilane sesquiterpenoids, paraconulones B−G (**3**−**8**), along with three known compounds, periconianone D (**9**), microsphaeropsisin (**10**), and 4-epi-microsphaeropsisin (**11**), were obtained from the marine-coastal-sediment-derived fungus, *Paraconiothyrium sporulosum* DL-16. Compounds **3** and **5** were the first examples of dimeric eremophilane sesquiterpenoids coupled through a C−C bond identified from microorganisms. The linkage of C-C bond in **3** was probably formed by a free radical coupling reaction, and the linkage in **5** could have been formed by a [2+2] cycloaddition reaction of two eremophilane units [25]. Compounds **3**−**6**, **8,** and **11** showed inhibitory effects on NO production in LPS-induced BV2 cells, with IC_50_ values of 6.9 ± 2.6, 7.7 ± 2.0, 2.8 ± 0.5, 8.1 ± 2.9, 8.1 ± 3.5, and 4.6 ± 3.5 µM, respectively, which were comparable with or better than the positive control, curcumin (IC_50_, 8.6 ± 1.6 μM). Compounds **7**, **9**, and **10** showed moderate or weak inhibitory effects on NO production in LPS-induced BV2 cells, with IC_50_ values of 40 ± 15, 98 ± 17, and 80 ± 38 µM, respectively [25]. Seven new sesquiterpene derivatives, AA03390 (**12**), eremofortin F (**13**), eremofortins G−J (**14**−**17**), and lithocarin A (**18**), were isolated from the mangrove endophytic fungus *Phomopsis* sp. SYSU-QYP-23 (East Harbour National Nature Reserve, Hainan Province, China). Compounds **12**–**18** showed potent inhibitory activities against NO production in LPS-induced RAW 264.7 cells, with IC_50_ values of 14.5, 13.5, 12.0, 8.6, 9.2, 13.5, and 10.5 μM, respectively. In particular, compounds **15** and **16** showed potent inhibitory activities against NO production in LPS-induced RAW 264.7 cells, with IC_50_ values of 8.6 and 9.2 μM, respectively. Compounds **12**–**14** and **17**–**18** exhibited moderate inhibitory activity compared to the positive control, NG-monomethyl-L-arginine (L-NMMA), with an IC_50_ value 15.0 μM. All the compounds showed no cytotoxic effects at the tested concentration [26]. Two undescribed drimane sesquiterpenes, ustusolates H (**19**) and I (**20**), were separated from a seagrass-derived fungus, *Aspergillus insuetus* SYSU6925 (Zhuhai, Guangdong, China). Compounds **19** and **20** exhibited potent anti-inflammatory activity by inhibiting the production of NO in RAW264.7 cells, with IC_50_ values of 21.5 and 32.6 μM, respectively [27]. Two new sesquiterpenes, **21** and **22**, were isolated from the mutant of the polar strain, *Eutypella* sp. D-1 (London Island of Kongsfjorden in Ny-Ålesund District, Arctic). Compounds **21** and **22** exhibited anti-inflammatory effects through inhibiting the release of NO from LPS-stimulated RAW264.7 cells. Furthermore, acorane-type sesquiterpene **22** could modulate the MAPK and NLRP3/caspase-1 signaling pathways and alleviate the CuSO_4_-induced systemic neurological inflammation symptoms in a transgenic fluorescent zebrafish model [28] (Figure 2).

#### 2.1.3. Diterpenoids

**Figure 3:**
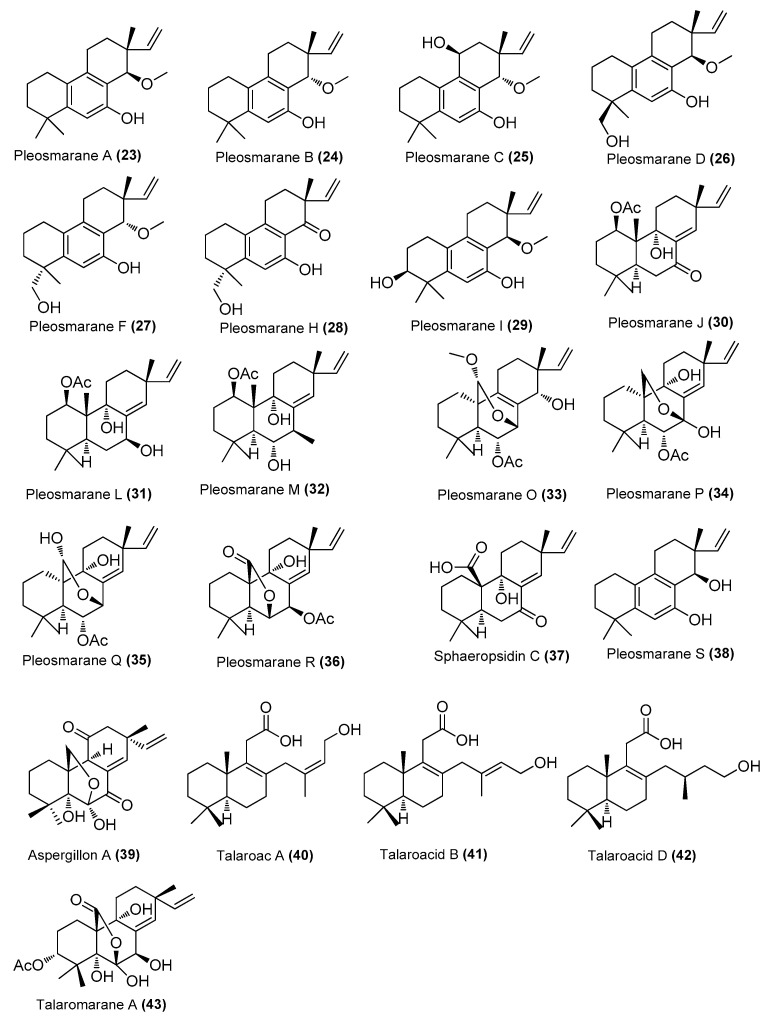
Anti-inflammatory diterpenoids produced by marine fungi (**23**–**43**).

Fourteen new isopimarane-type diterpenoids, pleosmaranes **A**–**D** (**23**–**26**), F (**27**), H–J (**28**–**30**), L (**31**), M (**32**), and O–R (**33**–**36**), together with two known analogs, sphaeropsidin C (**37**) and pleosmarane S (**38**), were found from the mangrove *Kandelia candel* endophytic fungus, *Pleosporales* sp. HNQQJ-1 (Dongzhai Harbor Mangrove Nature Reserve in Hainan Province, China). Compounds **23**–**29** possess an unusual aromatic B ring and a 20-nor-isopimarane skeleton. Compounds **33**–**35** contain a unique 2-oxabicyclo [2.2.2]octane moiety. Compound **36** features a rare 2-oxabicyclo [3.2.1]octane moiety. Compounds **23**–**38** showed potent anti-inflammatory activities, with IC_50_ values of 30, 37, 38, 42, 42, 19, 35, 33, 25, 35, 37, 30, 33, 35, 31, and 40 μM, respectively, compared with the positive control (L-NMMA, 33 μM). The preliminary structure–-activity relationship (SAR) of these isopimarane-type diterpenoids indicated that the carbonyl group at C-14 in **23**−**28** appeared to contribute to the NO-inhibitory activity, based on the comparison of the activities of **23**−**27** with those of **28**. The more potent activities of **23**, **24**, **26**, and **27** relative to those of **38** indicated that the methoxy group at C-14 was associated with enhancement of anti-inflammatory activity. Consequently, isopimarane diterpenoids have demonstrated significant potential as NO inhibitors and may be further explored as lead compounds for anti-inflammatory therapeutic applications [29]. The known diterpene, aspergillon A (**39**), was isolated from marine-derived fungus *Eutypella scoparia* GZU-4-19Y (Xuwen, Guangdong, China), which showed potent NO inhibition activity with IC_50_ value of 2.0 μM, and significantly suppressed the protein expression of iNOS and COX-2 at the concentration of 2.5 μM [30]. Three new diterpenes with 1,2,3,4,4a,5,6,8a-octalin skeletons, talaroacids A, B, and D (**40**−**42**), together with an isopimarane diterpenoid talaromarane A (**43**), were obtained from the mangrove endophytic fungus, *Talaromyces* sp. JNQQJ-4 (Jinniu Island Mangrove Nature Reserve, Guangzhou, China). Compound **43** contained a rare 2-oxabicyclo [3.2.1] octan moiety in isopimarane diterpenoids. Compound **41** had better anti-inflammatory activity than the positive control, quercetin (IC_50_, 11.33 μM), with an IC_50_ value of 4.59 μM. Compounds **40**, **42**, and **43** showed moderate anti-inflammatory activities, with IC_50_ values of 15.78, 21.60, and 13.38 μM, respectively. In addition, the better activity of **40** and **41** compared with **42** indicated that the ∆^14^ double bond in the side chain made a contribution to the NO-inhibitory activity. Compound **41** might be worthy of further study as a potential anti-inflammatory lead compound. None of the compounds were cytotoxic to RAW264.7 cells at the tested concentrations [31] (Figure 3).

#### 2.1.4. Meroterpenoids

**Figure 4:**
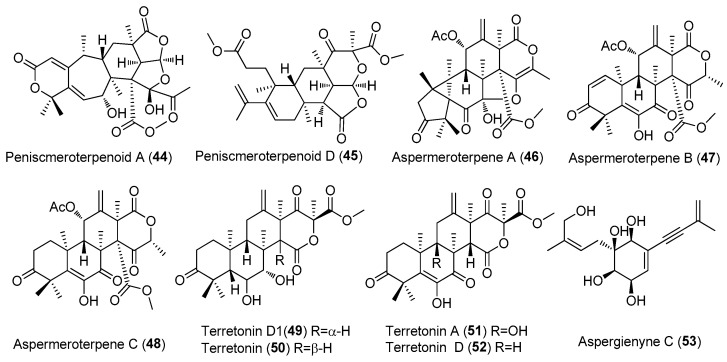
Anti-inflammatory meroterpenoids produced by marine fungi (**44**–**53**).

**Scheme 2:**
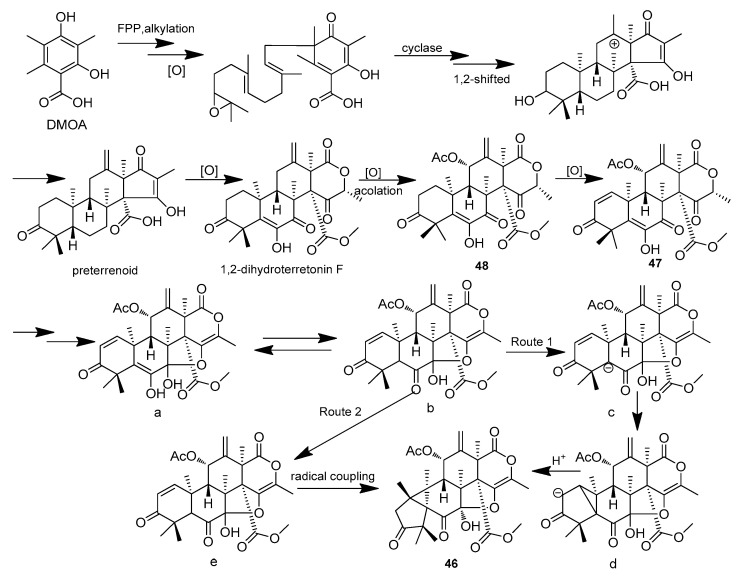
Plausible biogenetic pathway of **46**−**48** [33].

Two meroterpenoids, peniscmeroterpenoids A and D (**44** and **45**), were isolated from the marine *Onchidium* sp.-derived fungus, *Penicillium sclerotiorum* GZU-XW03-2. Compound **44** possessed an unprecedented and highly oxidized 6/7/6/5/5 pentacyclic system, featuring a unique tetrahydrofuro [2,3-b]furan-2(3H)-one motif. Compound **45** owned 6(*D*)/5(*E*) fused rings, which were not common in natural products. Compounds **44** and **45** inhibited the production of NO in RAW264.7 cells, with IC_50_ values of 26.60 ± 1.15 and 8.79 ± 1.22 μM, respectively. Moreover, compound **45** significantly suppressed the production of pro-inflammatory mediators (COX-2, IL-1*β* and IL-6) and the protein expression of the enzyme iNOS, with an IC_50_ value of 8.79 ± 1.22 μM [32]. Three new compounds, aspermeroterpene A–C (**46**–**48**), were isolated from the marine-derived fungus, *Aspergillus terreus* GZU 31-1. Their structures were elucidated by quantum chemical calculations, X-ray diffraction, and spectroscopic methods. Compound **46** had a highly congested 5/3/6/6/6/5 hexacyclic skeleton. The biogenetic pathway for **46**−**48** is proposed in Scheme 2 [33]. Compounds **46**−**48** showed potent inhibitory activity against LPS-induced NO production in RAW 264.7 cells, with IC_50_ values of 17.8, 14.1, and 13.4 μM, respectively, compared to the positive control (indomethacin, IC_50_ = 24.0 μM) [33]. A new meroterpenoid, terretonin D1 (**49**), and three known compounds, terretonin (**50**), terretonins A (**51**), and D (**52**), were isolated from marine *Pacific oyster*-derived fungus *Aspergillus terreus* ML-44 (Yangma lsland, Yantai, China). Compounds **49**−**52** inhibited the NO production of RAW264.7 macrophages stimulated by LPS, with inhibitory rates of 30.2%, 34.0%, 22.5% and 23.5%, respectively, at the concentration of 50 μg/mL [34]. One new undescribed diisoprenyl-cyclohexene-type meroterpenoid, aspergienyne C (**53**), was obtained from the mangrove *Kandelia cande* endophytic fungus *Aspergillus* sp. GXNU-Y65 (Beihai, China). Compound **53** significantly reduced the triglyceride (TG) content compared with fenofibrate (positive control) in palmitic acid + oleic acid-treated AML12 cells at the same concentration of 20 μM, and obviously increased the phosphorylation of acetyl-CoA carboxylase. No significant loss of cell viability was observed when the concentration of **53** was equal to or lower than 40 μM [35] (Figure 4).

### 2.2. Polyketides

Polyketides are a class of compounds distinguished by their structural diversity and a range of biological activities. They are synthesized by megaenzymes known as polyketide synthases (PKSs). These compounds were produced through a series of Claisen condensation reactions, typically employing acetyl-CoA, malonyl-CoA, and other substrates. A total of 110 anti-inflammatory polyketides have been isolated from marine-derived fungi, among which 60 are new compounds. Additionally, five of these new compounds (designated as **64**, **136**−**139**) possess novel skeleton structures.

#### 2.2.1. Lactones

**Figure 5:**
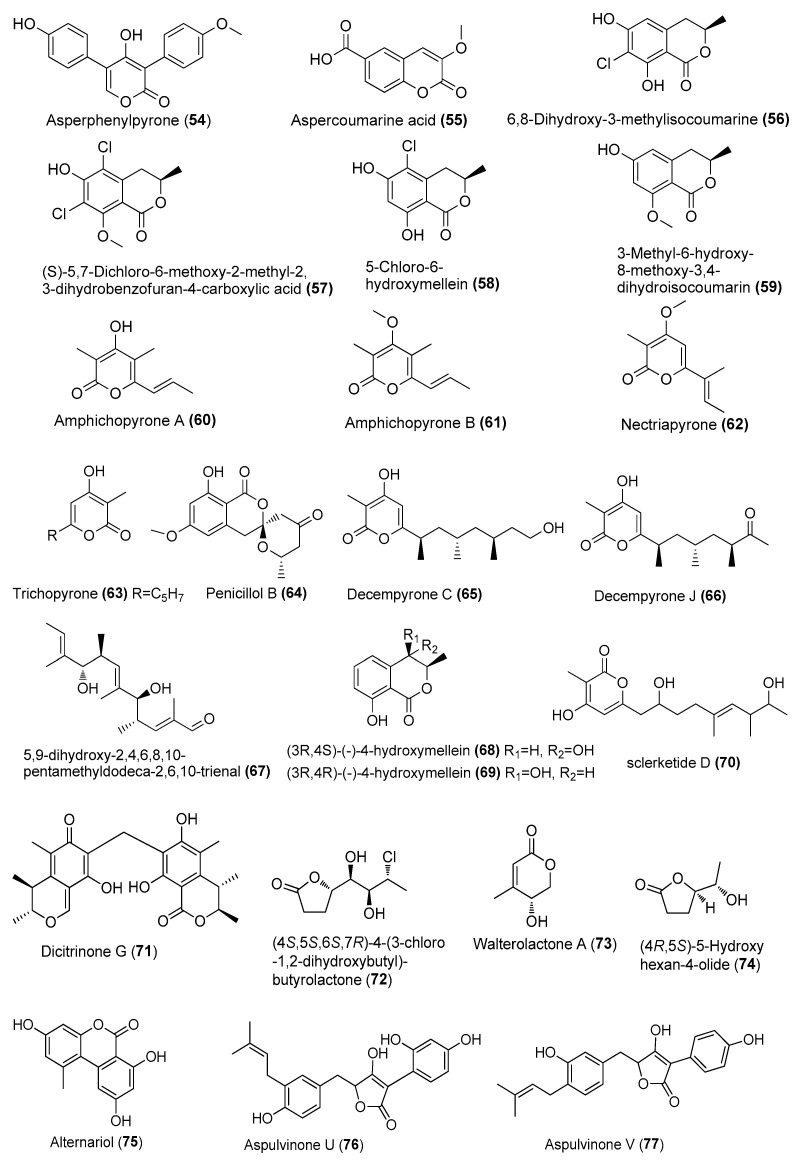

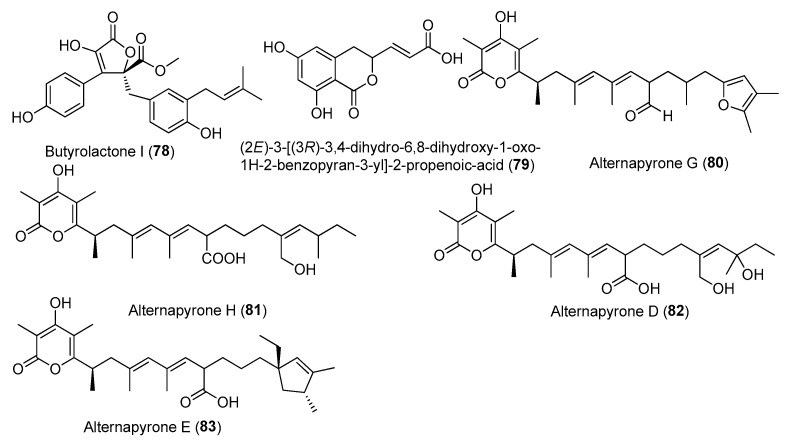
Lactones produced by marine fungi with anti-inflammatory activity (**54**–**83**).

Two new polyketides, asperphenylpyrone (**54**) and aspercoumarine acid (**55**), were isolated from the deep-sea sediment-derived fungus, *Aspergillus sydowii* MCCC 3A00324 (South Atlantic Ocean). Compounds **54** and **55** exhibited dose-dependent inhibitory effects against NO production induced by the LPS at the concentrations of 20 and 10 µM, respectively [24]. Four known polyketides, 6,8-dihydroxy-3-methylisocoumarine (**56**), (*S*)-5,7-dichloro-6-methoxy-2-methyl-2,3-dihydrobenzofuran-4-carboxylic acid (**57**), 5-chloro-6-hydroxymellein (**58**), and 3-methyl-6-hydroxy-8-methoxy-3,4-dihydroisocoumarin (**59**), were isolated from the mangrove *Avicennia marina* endophytic fungus *Amorosia* sp. SCSIO 4102 (Zhanjiang, Guangdong Province, China). Compounds **56**–**59** inhibited LPS-induced overproductions of NO and pro-inflammatory cytokines including IL-6, TNF-*α*, and MCP-1 in both mRNA and protein levels, with the condition that none of these compounds showed significant cytotoxicity. Compound **58** was identified as the most active compound, with strong anti-LPS-induced inflammation in RAW264.7 macrophages and in ALI mice, probably by inhibiting the PI3K/AKT signaling pathway. A preliminary SAR analysis was conducted, suggesting that the chlorine atom at C-5 and the hydrogen atom at C-7, together with the hydroxy group at C-8 in **58**, would probably increase the inflammatory activity, and the chlorine atom at C-7 may decrease the activity [36]. Two new *α*-pyrone derivatives, amphichopyrones A (**60**) and B (**61**), were obtained from the *Culturing ascidian*-derived fungus, *Amphichorda felina* SYSU-MS7908. The fungus was collected from the north atoll of the Xisha Islands in the South China Sea. Compounds **60** and **61** displayed potent anti-inflammatory activity by inhibiting NO production in RAW264.7 cells, with IC_50_ values of 18.09 ± 4.83 and 7.18 ± 0.93 µM, respectively [37]. Nectriapyrone (**62**) was isolated from the ascidian-derived fungus, *Diaporthe* sp. SYSU-MS4722, and showed anti-inflammatory activity with an IC_50_ value of 35.4 µM (positive control, indomethacin, IC_50_ = 35.8 µM) [38]. One known sorbicillinoid, trichopyrone (**63**), was identified from the mangrove *Hibiscus tiliaceus*-Linnn-derived fungus, *Penicillium* sp. DM815 (Wenchang, Hainan, China). It showed weakly inhibited LPS-induced NO release at 10 μM [39]. One new unique isocoumarin, penicillol B (**64**), featuring a spiroketal ring, was isolated from the barks of the mangrove *Avicennia marinav* (Forsk.) endophytic fungus, *Penicillium* sp. BJR-P2 (Yangjiang Hailing Island Mangrove Wetland Park, China). Its absolute configuration was presented based on ECD calculation, DP4+ analysis, and single-crystal X-ray diffraction. The condensation of one mole of acetyl coenzyme A with six moles of malonyl coenzyme A results in the formation of a linear polyketide chain. Subsequent steps, including keto-reduction, cyclization, methylation, and hydroxylation lead to the production of **64** [40]. Compound **64** inhibited LPS-induced NO production in RAW 264.7 cells with an IC_50_ value of 12 µM, being more potent than the positive control, indomethacin (IC_50_, 35.8 ± 5.7 µM) [40]. One new polypropionate derivative, decempyrone C (**65**), and one known analogue, decempyrone J (**66**), were isolated from the marine sea-grass-derived fungus, *Fusarium decemcellulare* SYSU-MS6716 (Lingshui Xincungang and Li’angang Special Protected Area, Hainan Province, China). The *J*-based configurational analysis (JBCA), chemical degradation, and geminal proton rule were adopted to determine the absolute configurations in the side chain. Compounds **65** and **66** exhibited potent anti-inflammatory activity by inhibiting the production of NO in RAW264.7 cells activated by LPS, with IC_50_ values of 22.4 ± 1.8 and 21.7 ± 1.1 μM, respectively [41]. One new linear polyketide, 5,9-dihydroxy-2,4,6,8,10-pentamethyldodeca-2,6,10-trienal (**67**), and two polyketides, (3*R*,4*S*)-(-)-4-hydroxymellein (**68**) and (3*R*,4*R*)-(−)-4-hydroxymellein (**69**), were isolated from the alga *Hypnea pannosa*-derived fungus, *Aspergillus ochraceopetaliformis* SCSIO 41020 (South China Sea in Luhuitou, Hainan Province, China). Compounds **67**–**69** possessed inhibitory activity against the excessive production of NO and pro-inflammatory cytokines in LPS-treated RAW 264.7 macrophages in a dose-dependent manner without cytotoxicity. The preliminary SAR was discussed, indicating that the hydroxy group at C-9 in **67** played a pivotal role in NO inhibition. Moreover, the 4*R* configuration in **69** probably increased the inflammatory activity. Further studies revealed that compound **67** was active in blocking the release of pro-inflammatory cytokines (IL-6, MCP-1, and TNF-*α*) induced by LPS both in vivo and in vitro. These findings provided a basis for the further development of linear polyketides as promising anti-inflammatory agents [42]. One new *α*-pyrone, sclerketide D (**70**), was isolated from the gorgonian-derived fungus, *Penicillium sclerotiorum* CHNSCLM-0013 (Weizhou coral reef in South China Sea). Compound **70** exhibited significant inhibitory activity against the NO production in the LPS-induced macrophage, RAW 264.7, with an IC_50_ value of 5.5 μM [43]. Dicitrinone G (71), a novel citrinin dimer derived from the marine fungus, *Penicillium* sp. GGF 16-1-2, has been shown to suppress pancreatic angiogenesis by modulating the activation of the NLRP3 inflammasome. Furthermore, in both in vivo and in vitro studies, it has demonstrated the ability to inhibit pancreatic cancer angiogenesis by regulating the inflammatory microenvironment through IL-18. These findings suggested that **71** could impede angiogenesis through the NLRP3/IL-18 pathway and may possess the potential to curb tumor progression [44]. A new chloro-containing *γ*-butyrolactone derivative, (4*S*,5*S*,6*S*,7*R*)-4-(3-chloro-1,2-dihydroxybutyl)-butyrolactone (**72**), was isolated from the fruits of the mangrove plant *Sonneratia glauca* endophytic fungus *Neofusicoccum parvum* Y2NBKZG1016 (Nansha District, Guangzhou, China). Compound **72** exhibited weak anti-inflammatory activity at the concentration ≥ 6.25 μM, reaching a maximal inhibition rate of 28.9%, and had no cytotoxicity to RAW 264.7 cells [45]. Two known compounds, walterolactone A (**73**) and (4*R*,5*S*)-5-hydroxyhexan-4-olide (**74**), were obtained from the deep-sea sulfide-sample-derived fungus, *Samsoniella hepiali* W7 (South Atlantic), by using a molecular networking approach. Compounds **73** and **74** showed potent inhibitory activity against NO production in LPS-activated BV-2 microglia cells, with inhibition rates of 38.6% and 58.2%, respectively, at the concentration of 1 µM. Compound **74** exhibited remarkable inhibitory activity, with an IC_50_ value of 426.2 nM [46]. Alternariol (**75**) was isolated from the Antarctic fungal strain, *Pleosporales* sp. SF-7343 (King George Island, Antarctica). It could inhibit the secretion of IL-8 and IL-6 in tumor necrosis factor-*α*/interferon-*γ*-treated HaCaT cells in an inflammatory disease, atopic dermatitis [47]. Two new polyketides, aspulvinones U (**76**) and V (**77**), were isolated from the marine alga *Ulva lactuca* L.-derived fungus, *A. terreus* NTU243, collected from the northeastern coast of Taiwan, China. Compound **77** inhibited 45.0% of the production of NO under a concentration of 10 μM without any cytotoxicity. Compounds **76** and **77** inhibited LPS-induced MMP-9-mediated gelatinolysis (56.0% and 67.8%, respectively) compared the vehicle-treated condition [48]. The known compound, butyrolactone I (**78**), was separated from the mangrove plant *Acanthus illicifolius*-derived fungus, *Aspergillus flavipes* MTCC 5220 (Goa, India). It blocked IL-6 and TNF-*α* production in LPS-activated THP-1 cells, with IC_50_ values of 2.69 ± 0.5 µM and 6.64 ± 0.4 µM, respectively, and exhibited inhibition activity, with IC_50_ values of 12.03 ± 0.85 µM (IL-6) and 43.29 ± 0.76 µM (TNF-*α*), with low toxicity to host cells in LPS-stimulated THP-1 cells [49]. Furthermore, **78** was also isolated from the coral *Porites pukoensis*-derived fungus, *Aspergillus terreus* XWC21-10 (Zhanjiang seawaters in the South China Sea), significantly reduced NO production in LPS-induced BV2 microglia cells, and also inhibited the expression of iNOS and COX-2. Additionally, **78** suppressed LPS-induced phosphorylation and nuclear translocation of NF-*κ*B in BV2 microglia cells. A docking study showed that molecular events occurred at the binding interface of **78** with NF-*κ*B and COX-2 interaction sites, indicating that **78** may act as a potential candidate for treating inflammation-related neurological disorders and provide a new insight into the secondary metabolism derived from marine fungi [50]. Furthermore, its anti-inflammatory and intestinal-barrier-protective effects were assessed using the LPS-induced IPEC-J2 inflammation model and the DSS-induced IBD model in mice. It was found that **78** alleviated inflammatory responses by TLR4/NF-*κ*B and the MAPK signal pathway, according to in vitro and in vivo studies. Therefore, **78** might potentially be used as an ocean drug to prevent intestinal bowel disease [50]. Furthermore, the modulation of the gut microbiota of **78** was demonstrated to be one of the mechanisms contributing to anti-inflammation properties and improving endoxemia [51]. One new polyketide, (2*E*)-3-[(3*R*)-3,4-dihydro-6,8-dihydroxy-1-oxo-1H-2-benzopyran-3-yl]-2-propenoic-acid (**79**), was isolated from the deep-sea hydrothermal vent sediment-derived fungus, *Penicillium* sp. TW58-16 (Kueishantao, Taiwan, China). Compound **79** suppressed NO production in LPS-stimulated macrophages [52]. Two new linear polyketides, alternapyrones G (**80**) and H (**81**)**,** and two known 6-alkenylpyrone polyketides, alternapyrones D (**82)** and E (**83),** were found in a marine *Phakellia fusca*-derived fungus, *Arthrinium arundinis* ZSDS-F3 (Xisha Islands of China). The biosynthetic gene cluster (alt) for alternapyrones was identified from *A. arundinis* ZSDS-F3 and validated by heterologous expression in *A. nidulans* A1145 ΔSTΔEM. Compared with the vehicle control, the ratio at which compounds **80**–**83** inhibited NO release was above 50% at the concentration of 20 μM. Compound **80** evidently suppressed TNF-*α* and IFN-*γ* production, decreased LPS-induced IL-4 and IL-10 at concentrations of 2 and 20 μM, respectively, and significantly suppressed the production of NO and the mRNA expression of iNOS (M1 marker) at the concentration of 20 μM. Compound **80** not only suppressed M1 polarization in LPS-stimulated BV2 microglia but also stimulated dendrite regeneration and neuronal survival after A*β* treatment, suggesting that alternapyrone G could be employed as a privileged structure for the development of Alzheimer’s disease treatments [53] (Figure 5).

#### 2.2.2. Azaphilones

**Figure 6:**
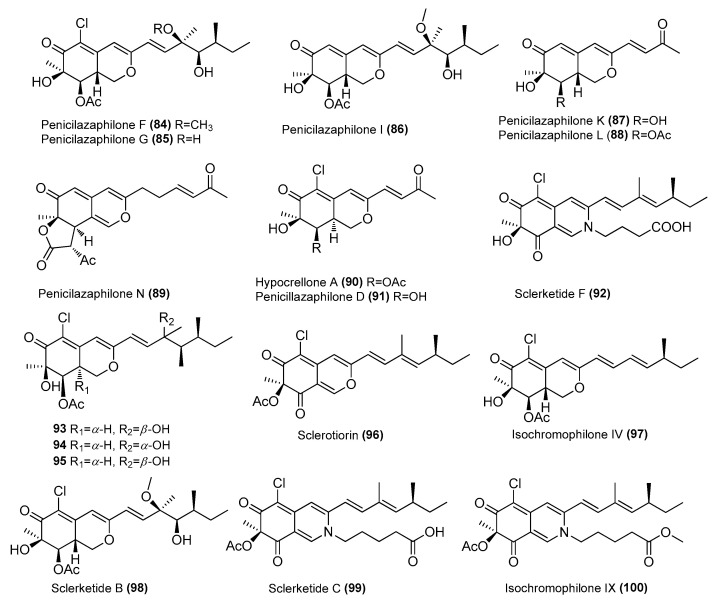
Anti-inflammatory azaphilones produced by marine-derived fungi (**84**–**100**).

Six new azaphilones, including penicilazaphilones F (**84**), G (**85**), I (**86**), K (**87**), L (**88**), and N (**89**), together with two known analogs, hypocrellone A (**90**) and penicillazaphilone D (**91**), were isolated from the culture of the sponge *Holoxea* sp.-derived fungus, *Penicillium sclerotiorum* E23Y-1A (Quanfu Island, Hainan, China). Compounds **84**, **85**, **90**, and **91** inhibited the production of NO in LPS-induced BV-2 cells, with IC_50_ values of 31.7 ± 1.5, 34.5 ± 1.4, 25.3 ± 2.2, and 34.8 ± 1.9 μM, respectively. Compound **89** exhibited moderate inhibition of NO production in LPS-stimulated RAW264.7 cells, with an IC_50_ value of 22.63 ± 2.95 µM, and the other compounds exhibited weak inhibition. At the same time, these compounds showed no obvious cytotoxicity at the concentration of 50.0 μM [54,55]. One new sclerotioramine, sclerketide F (**92**), was isolated from the mangrove *Bruguiera gymnorhiza* endophytic fungus, *Penicillium sclerotiorin* SCNU-F0040 (Zhanjiang Mangrove Nature Reserve, Guangdong Province, China). Compound **92** showed moderate COX-2 inhibitory activity, with an IC_50_ value of 47.8 µM [56]. Two new azaphilones, 8a-epi-hypocrellone A (**93**) and 8a-epi-eupenicilazaphilone C (**94**), together with three known azaphilones, hypocrellone A (**95**), sclerotiorin (**96**) and isochromophilone IV (**97**), were obtained from the alga *Grateloupia* sp.-derived fungus, *Penicillium sclerotiorum* (Yilan County, Taiwan, China). Compounds **93**, **95**, and **97** inhibited TNF-*α*-induced NF-*κ*B phosphorylation but did not change the NF-*κ*B activity at the concentration of 20 µM. Compounds **94** and **96** respectively promoted and inhibited SMAD-mediated transcriptional activities stimulated by transforming growth factor-*β* (TGF-*β*). Compound **94** could promote both TGF-*β*/Smad signaling and transcriptional function. Compound **96**, similarly to the selective TGF*β*RI kinase inhibitor, LY3200882, inhibited both TGF-*β*/Smad-mediated signaling and transcriptional function [57]. Two new azaphilone analogous sclerketides, B (**98**) and C (**99**), together with one known compound, isochromophilone IX (**100**), were isolated from the gorgonian-derived fungus, *Penicillium sclerotiorum* CHNSCLM-0013 (Weizhou coral reef in South China Sea). Compounds **98**–**100** exhibited significant inhibitory activities against NO production in the LPS-induced macrophage RAW 264.7, with IC_50_ values of 3.4, 17.6, and 2.7 μM, respectively [43] (Figure 6).

#### 2.2.3. Xanthones

**Figure 7:**
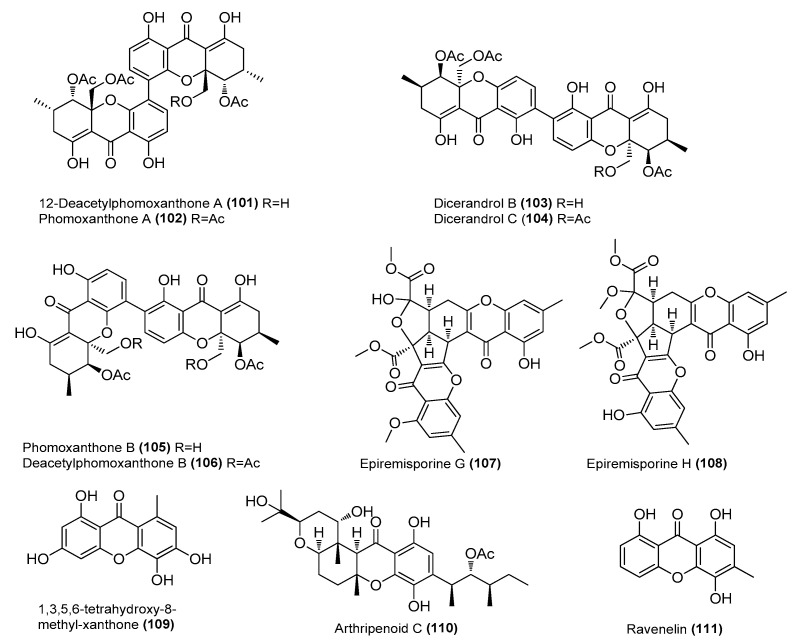
Anti-inflammatory xanthones produced by marine-derived fungi (**101**–**111**).

Six known dimeric xanthones, 12-deacetylphomoxanthone A (**101**), phomoxanthone A (**102**), dicerandrol B (**103**), dicerandrol C (**104**), phomoxanthone B (**105**), and deacetylphomoxanthone B (**106**), were obtained from the ascidian-derived fungus, *Diaporthe* sp. SYSU-MS4722 (Bay of Da’ao, Guangdong Province, China). Compounds **101**−**106** showed strong inhibition of NO production in RAW264.7 cells activated by LPS, with IC_50_ values 6.3, 7.5, 6.3, 7.6, 8.0, and 7.8 µM, respectively, which was better than the positive control, indomethacin (IC_50_ = 35.8 µM) [58]. Two new chromone analogs, epiremisporines G (**107**) and H (**108**), were isolated from marine-origin *Penicillium citrinum* (Hazailiao, Dongshi, Chiayi, Taiwan, China). Compounds **107**−**108** remarkably suppressed N-formyl-L-methionyl-L-leucyl-L-phenylalanine (fMLP)-induced superoxide anion generation by human neutrophils, with IC_50_ values of 31.68 ± 2.53 and 33.52 ± 0.42 µM, respectively, while the positive control, ibuprofen, had an IC_50_ value of 28.56 µM [59]. Two known compounds, 1,3,5,6-tetrahydroxy-8-methyl-xanthone (**109**) and arthripenoid C (**110**), were isolated from the sea-anemone-derived fungus, *Arthrinium arundinis* MA30 (sea shore of Badouzi). Compounds **109** and **110** demonstrated distinctive inhibitory activities against NO production in mouse microglial BV-2 cells, with IC_50_ values of 5.3 ± 0.6 and 1.6 ± 0.4 μM, respectively, and showed no significant cytotoxicity [60]. One known xanthone, ravenelin (**111**), isolated from the mangrove endophytic fungus, *Setosphaeria rostrata* (Prachuap Kiri Khan, Thailand), displayed significant activity, with an IC_50_ value of 6.27 μM, and could suppress iNOS and COX-2 expression [61] (Figure 7).

#### 2.2.4. Other Polyketides

**Figure 8:**
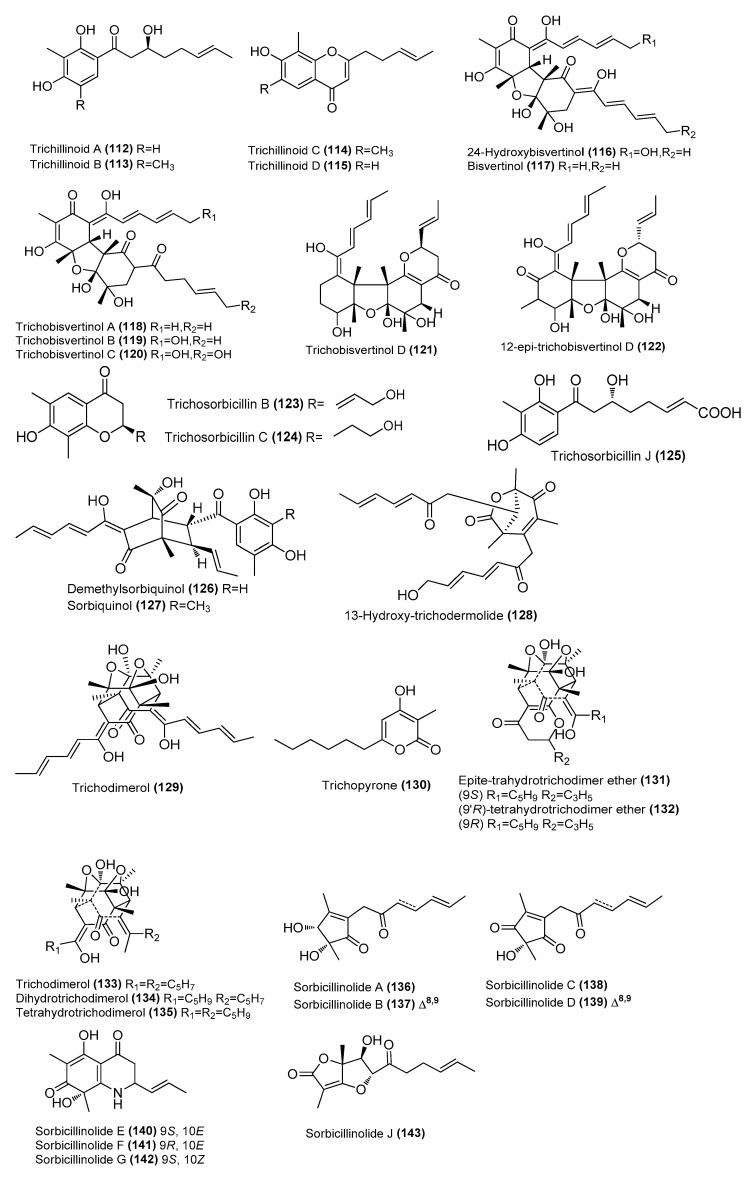

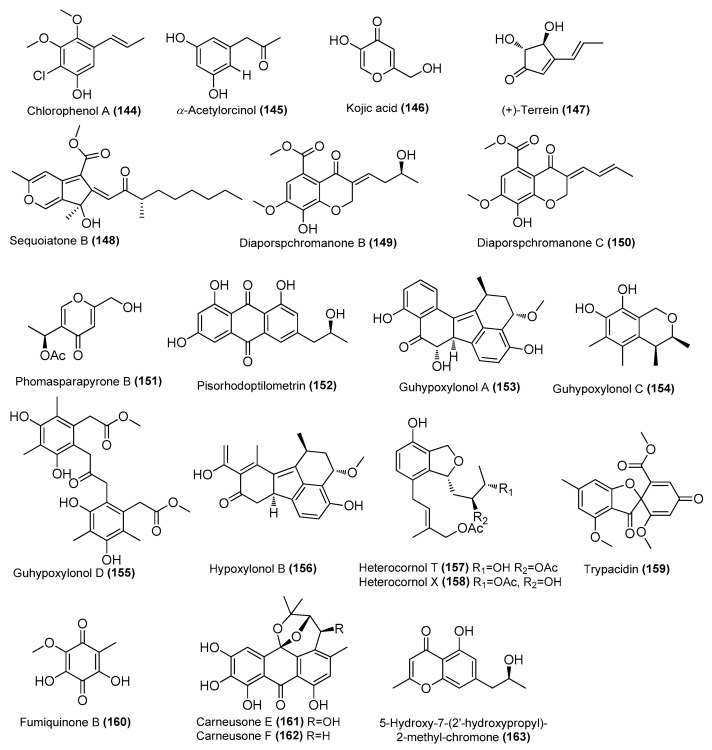
Other polyketides produced by marine fungi with anti-inflammatory activity (**112**–**163**).

**Scheme 3:**
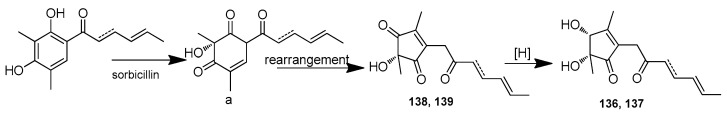
Postulation of the biogenetic relationships of **136**–**139** [36].

Four new monomeric sorbicillinoids, trichillinoids A–D (**112**−**115**), were obtained from the marine fish *Sebastes schlegelii*-derived fungus, *Trichoderma* sp. G13 (Yangma Island, Yantai, China). Compounds **112**−**115** showed strong inhibition of NO production in RAW264.7 cells activated by LPS, with IC_50_ values of 14, 14, 16, and 20 µM, respectively, compared with the positive control, dexamethasone (IC_50_ = 10 µM) [62]. Six new dimeric sorbicillinoids, 24-hydroxybisvertinol (**116**), trichobisvertinols A−D (**118**−**121**), and 12-epi-trichobisvertinol D (**122**), two new monomeric sorbicillinoids, trichosorbicillins B (**123**) and C (**124**), along with one known analogue, bisvertinol (**117**), were isolated from the marine-sponge-derived fungus, *Trichoderma reesei* 4670 (Shantou, Guangdong Province, China). Compounds **121** and **122** were the first examples of bisorbicillinoids possessing a benzofuro [2,3-h] chromene scaffold from a natural source; a similar structure has been synthesized by the Gulder group. Compounds **116**−**124** exhibited potent anti-inflammatory activity by inhibiting the production of NO in RAW264.7 cells activated by LPS, with IC_50_ values 6.1, 9.9, 5.9, 22, 24, 22, 32, 8.5, and 38 μM, respectively. A preliminary SAR analysis indicated that the anti-inflammatory activities of the sorbicillinoids mainly depended on the structural types and the functional groups of the sorbyl side chain. For monomeric sorbicillinoids, the double bonds of the sorbyl side chain played an important role in their anti-inflammatory action, as **123**, with at least one double bond in the side chain, was much more active than **124**, without a double bond in the side chain [63]. Two new sorbicillinoids, trichosorbicillin J (**125**) and demethylsorbiquinol (**126**), together with three known compounds, sorbiquinol (**127**), 13-hydroxy-trichodermolide (**128**), and trichodimerol (**129**), were isolated from the mangrove *Avicennia marina*-derived fungus, *Trichoderma reesei* BGRg-3 (Guangdong Province, China). Compounds **128** and **129** presented remarkable anti-inflammatory activities, with 47% and 67% inhibition of IL-6, and 85% and 87% inhibition of IL-1*β*, respectively, which was even more effective than the positive control (dexamethasone) at the concentration of 25 µM. Furthermore, compounds **126** and **127** showed potent effects, with stronger inhibition than dexamethasone on IL-1*β* at the same concentration. Compounds **125**–**127** also showed potent inhibition of IL-6 (45%, 27%, and 35%%, respectively) and IL-1*β* (21%, 75%, and 58%, respectively) [64]. Six known sorbicillinoids, trichopyrone (**130**), epite-trahydrotrichodimer ether (**131**), (9′*R*)-tetrahydrotrichodimer ether (**132**), trichodimerol (**133**), dihydrotrichodimerol (**134**), and tetrahydrotrichodimerol (**135**), were isolated from the mangrove *Hibiscus tiliaceus*-Linnn-derived fungus, *Penicillium* sp. DM815 (Qinglan, Wenchang, Hainan Province, China). Compounds **130**–**135** weakly inhibited LPS-induced NO release at 10 μM. Compounds **131** and **135** both dose-dependently inhibited the LPS-induced expression of iNOS, although the effect of **135** was much stronger than that of **131**. Compound **135** significantly inhibited LPS-induced NO production in RAW264.7 cells by inhibiting the upregulation of iNOS expression in a dose-dependent mode, and it did not exhibit inhibition of cell survival, even at the concentration of 20 μM, indicating that it is not toxic to cells [39]. Eight undescribed sorbicilinoids, sorbicillinolides A–G (**136**–**142**) and sorbicillinolide J (**143**), were determined by using the chemical fingerprinting approach utilizing LC-MS/MS coupled with 2D NMR data from the deep-sea sediment-derived fungus, *Penicillium rubens* F54 (Pacific Ocean). The cyclopentenone core of **136**–**139** was derived from sorbicillin/dihydrosorbicillin through a new oxidative rearrangement. Biogenetically, **136**–**139** are believed to originate from sorbicillin and dihydrosorbicillin. The oxidation of sorbicillin/dihydrosorbicillin produces an intermediate a, and then this intermediate undergoes cleavage of the C-5/C-6 bond, resulting in rearrangement to form a cyclopentendione nucleus **138**–**139**. The reduction of a ketone to a hydroxy group in the nucleus leads to the formation of **136** and **137** (Scheme 3) [36]. Moreover, the epoxidation of sorbicillin yields an intermediate b, which incorporates an amine unit (the pathway remains unclear). The stereoisomers of **140**–**142** incorporate a nitrogen unit, forming a unique hydroquinoline nucleus. This amine-incorporated intermediate then proceeds through nucleophilic ring cyclization, resulting in the generation of **140**–**142**, with compound **142** probably derived from **140** through olefinic isomerization. Further bioassays involving non-cytotoxic analogues against LPS-stimulated BV2 cells revealed that **136**–**143** at 10 μM exhibited inhibitory effects on NO and PGE2 production, with inhibition rates of 68.6%, 36.6%, 64.7%, 44.5%, 54.9%, 41.9%, 44.5%, and 33.4%, respectively. Notably, analogues **140** and **142** showed more potent inhibition against NO production than L-NMMA (methylarginine acetate), an NOS inhibitor, with IC_50_ values of 6.6 and 6.9 μM, respectively. Compounds **136** and **138** exhibited significant anti-neuroinflammation in LPS-stimulated BV-2 macrophages, achieved by the potent inhibition of NO and PGE2 production through the interruption of the RNA transcription of iNOS, COX-2, and IL-6 in the NF-*κ*B signaling pathway. Further investigation identified COX-2 as a potential target of **136**, suggesting that **136** is a potential lead compound for the development of a non-steroidal anti-neuroinflammatory agent [65]. One new propenylphenol derivative, chlorophenol A (**144**), and two known compounds, *α*-acetylorcinol (**145**) and kojic acid (**146**), were isolated from the mangrove *Avicennia marina* endophytic fungus, *Amorosia* sp. SCSIO 4102 (Zhanjiang, Guangdong Province, China). Compounds **144**–**146** inhibited the LPS-induced overproduction of NO and pro-inflammatory cytokines, including IL-6, TNF-*α*, and MCP-1, in both mRNA and protein levels, with the condition that none of these compounds showed significant cytotoxicity [36]. One known compound, (+)-terrein (**147**), was isolated from the marine alga *Ulva lactuca*-derived fungus, *A. terreus* NTU243, collected from the northeastern coast of Taiwan, and also from the marine mangrove plant *Acanthus illicifolius*-derived fungus, *Aspergillus flavipes* MTCC 5220 (Goa, India). Compound **147** inhibited 49.2% of the NO production under the concentration of 10 μM without any cytotoxicity, and it exhibited IL-6 and TNF-*α* inhibition activity, with IC_50_ values of 8.5 ± 0.68 and 15.76 ± 0.18 µM, respectively [48]. The known compound, sequoiatone B (**148**), isolated from the gorgonian-derived fungus, *Penicillium sclerotiorum* CHNSCLM-0013 (Weizhou coral reef in South China Sea), exhibited significant inhibitory activity against NO production in the LPS-induced macrophage, RAW 264.7, with an IC_50_ value of 5.2 μM [43]. Two new chromone compounds, diaporspchromanones B (**149**) and C (**150**), were separated from the mangrove-derived fungus, *Diaporthe* sp. XW12-1 (Xuwen, Guangdong Province, China). Compounds **149** and **150** possessed a 3-substituted-chroman-4-one skeleton, which is rarely found in natural sources, and showed potent anti-inflammatory effects, with IC_50_ values of 19.06 ± 3.60 and 9.56 ± 0.18 μM, respectively, which was better than the positive control, indomethacin (IC_50_ = 70.33 ± 0.95 μM) [66]. One new pyrone derivative, phomasparapyrone B (**151**), was isolated from marine mangrove *Acanthus ilicifolius* endophytic fungus *Phomopsis asparagi* LSLYZ-87 (Huizhou Mangrove National Nature Reserve, Guangdong Province, China). Compound **151** showed moderate inhibition of NO accumulation induced by LPS on BV-2 cells in a dose-dependent manner at 30, 40, and 50 μM, and without cytotoxicity, at a concentration of 50.0 μM [67]. One new anthraquinone, pisorhodoptilometrin (**152**), was isolated from the sponge-associated fungal strain, *Penicillium oxalicum* CLC-MF05 (Cu Lao Cham islands, Quang Nam, Vietnam). Compound **152** inhibited the LPS-induced production of NO in BV-2 cells, with an IC_50_ value of 15.2 ± 0.8μM, and showed an inhibitory effect on the overproduction of the pro-inflammatory mediators NO and PGE2, the overexpression of iNOS and COX-2, and the mRNA overexpression of the pro-inflammatory cytokines TNF-*α*, IL-1*β*, IL-6, and IL-12 in LPS-stimulated BV2 and rat primary microglia. The inhibitory effect of **152** was found to be regulated by the inactivation of the NF-*κ*B, MAPK, and TLR4/MyD88 signaling pathways, indicating that **152** presented potential anti-inflammatory candidates for the treatment of neurodegenerative diseases [58]. Three new polyketides, guhypoxylonols A (**153**), C (**154**), and D (**155**), and one known compound, hypoxylonol B (**156**), were isolated from the mangrove *Acanthus ilicifolius* endophytic fungus, *Aspergillus* sp. GXNU-Y45 (Beihai City, China). Compounds **153**–**156** showed inhibitory activity against the production of NO, with IC_50_ values of 14.42 ± 0.11, 18.03 ± 0.14, 16.66 ± 0.21, and 21.05 ± 0.13 µM, respectively [68]. Two new polyketide derivatives, heterocornols T (**157**) and X (**158**), were isolated from the sponge *Phakellia fusca*-derived fungus *Pestalotiopsis heterocornis* XWS03F09 (Xisha Islands, China) by one strain–many compounds (OSMAC) manipulation. Compounds **157** and **158** significantly inhibited the production of LPS-induced NO in RAW 264.7 cells with no cytotoxicity, compared to the positive drug, dexamethasone (DXM, IC_50_ = 33 μM), and markedly suppressed the iNOS protein expression in LPS-induced RAW 264.7 cells in a concentration-dependent manner. The findings indicated that the two novel polyketide derivatives could potentially serve as promising candidates for anti-inflammatory activity [69]. Two known polyketides, trypacidin (**159**) and fumiquinone B (**160**), were isolated from the cold-seep-derived fungus, *Talaromyces helicus* SCSIO41311 (South China Sea). Compound **160** showed more potent NO-inhibitory activity (IC_50_ = 9.65 μM) than eicosapentaenoic acid (EPA), with an IC_50_ value of 15.54 μM. Compound **160** showed moderate NO-inhibitory effects, with an IC_50_ value of 38.62 μM [70]. Two new benzophenone derivatives, carneusones E (**161**) and F (**162**), were isolated from the sponge-derived fungus, *Aspergillus carneus* GXIMD00543 (Weizhou islands coral reef, Beibu Gulf, China). Compounds **161** and **162** exhibited moderate anti-inflammatory effects on NO secretion when using LPS-induced RAW 264.7 cells, with EC_50_ values of 34.6 ± 0.9 and 20.2 ± 1.8 μM, respectively [71]. One new compound, 5-hydroxy-7-(2′-hydroxypropyl)-2-methyl-chromone (**163**), was isolated from the sponge-associated fungus, *Penicillium oxalicum* CLC-MF05 (Cu Lao Cham islands, Quang Nam, Vietnam). Compound **163** inhibited the LPS-induced production of NO in BV-2 cells, with an IC_50_ value of 75.5 ± 3.8 μM, and showed an inhibitory effect on the overproduction of PGE2, the overexpression of iNOS and COX-2, and the mRNA overexpression of TNF-*α*, IL-1*β*, IL-6, and IL-12 in LPS-stimulated BV2 and rat primary microglia. The inhibitory effect of **163** was regulated by the inactivation of the NF-*κ*B, MAPK, and TLR4/MyD88 signaling pathways, indicating that **163** is potential anti-inflammatory candidate for the treatment of neurodegenerative diseases [72] (Figure 8).

### 2.3. Nitrogen-Containing Compounds

Nitrogenous secondary metabolites are prevalent in nature and exhibit a diverse array of biological activities. A comprehensive study of marine-derived fungi led to the discovery of 76 nitrogen-containing compounds, among which 35 are novel. These compounds encompass 67 alkaloids and 9 peptides. Among the 35 new compounds, four (designated as **221**, **222**, **256**, and **257**) were identified as possessing novel skeletal structures.

#### 2.3.1. Alkaloids

**Figure 9:**
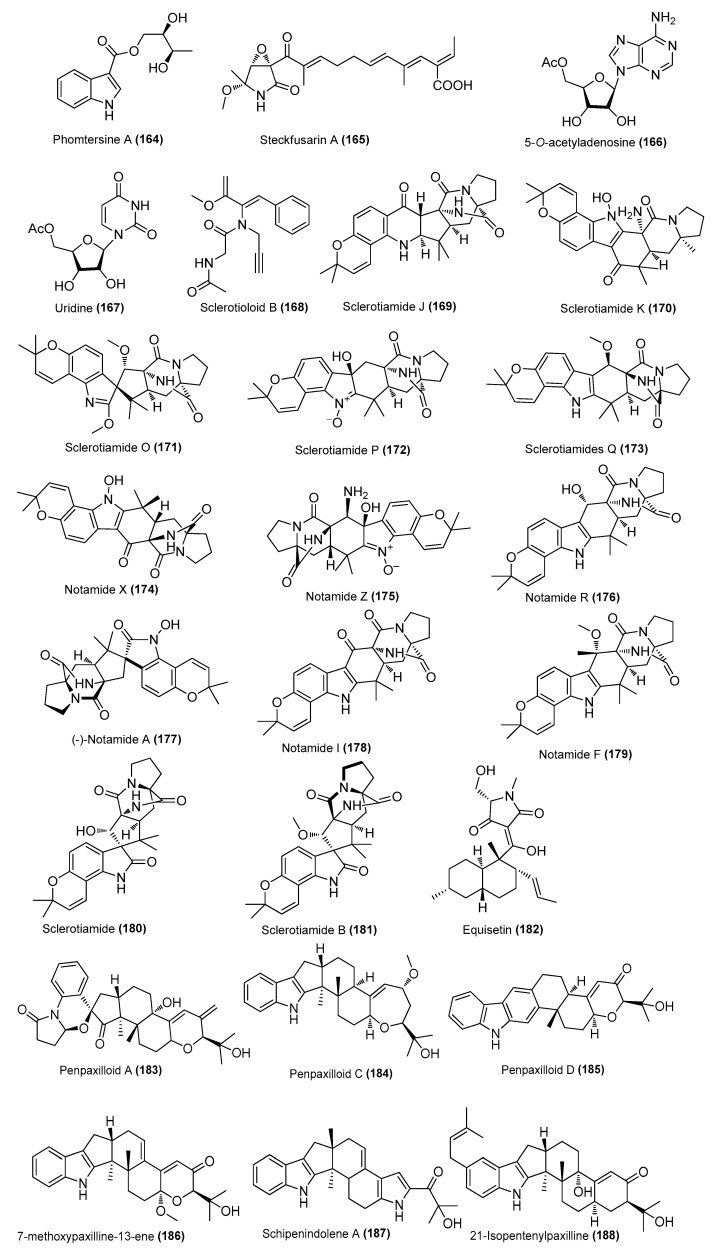

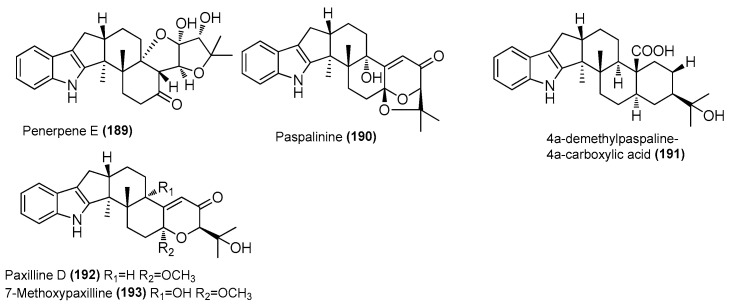
Alkaloids produced by marine-derived fungi with anti-inflammatory activity (**164**–**193**).

One new 3-carboxy-indole derivative, phomtersine A (**164**), was isolated from the marine-sediment-derived fungus, *Phomopsis tersa* FS441 (at a depth of 3000 m in the Indian Ocean). The structure was sufficiently established by extensive 1D and 2D NMR techniques and the modified Snatzke’s method. The derivative exhibited moderate inhibitory activity against LPS-induced NO production, with an IC_50_ value of 83.57 ± 2.81μM [73]. Steckfusarin A (**165**), a new fusarin derivative, isolated and identified from the green algae, *Botryocladia* sp. fungus *Penicillium steckii* SCSIO 41040 (South China Sea), showed weak anti-inflammatory activity at a concentration of 20 µM [74]. Two known miscellaneous compounds, 5-*O*-acetyladenosine (**166**) and 5-*O*-acetyluridine (**167**), were obtained from the deep-sea sulfide-sample-derived fungus, *Samsoniella hepiali* W7 (South Atlantic), by using the molecular networking approach. Compounds **166** and **167** showed potent inhibitory activity against NO production in LPS-activated BV-2 microglia cells, with inhibition rates of 34.2% and 30.7%, respectively, at a concentration of 1 µM [46]. One new alkaloid, sclerotioloid B (**168**), was obtained under the guidance of MS/MS-based molecular networking from the marine-derived fungus, *Aspergillus sclerotiorum* ST0501 (Guangdong, China). This alkaloid showed inhibition of NO production induced by LPS, with an inhibition rate that was 28.92% than that of dexamethasone (25.87%) [75]. Ten undescribed notoamidetype alkaloids, namely sclerotiamides J, K, and O−Q (**169**−**173**), and eight known compounds, notamide X (**174**), notamide Z (**175**), notamide R (**176**), (-)-notamide A (**177**), notamide I (**178**), notamide F (**179**), sclerotiamide (**180**), and sclerotiamide B (**181**), were isolated from a marine gorgonian-derived fungus, *Aspergillus sclerotiorum* LZDX-33-4, in the South China Sea. Compounds **169**–**181** possessed inhibitory effects against LDH and IL-1*β* expression in BV-2 cells. The bioassay results demonstrated that analogs **170**, **172**–**173**, **176**, and **179** significantly down-regulated the expression of LDH and IL-1*β* in BV-2 cells with more than 50% inhibition at a concentration of 10 μM. The preliminary analyses of the SAR indicated that the active analogs of **172**, **173**, **176**, and **179** are characterized by a 6,6,5,6,6,5-hexacyclic scaffold with mono-substitution of the OH or MeO group at C-18 or C-19, whereas **178**, with 18-hydroxylation and 19-methoxylation, and **170**, **174**, and **178,** with a ketone at C-19, attenuated the activities. Analogs bearing a spiro-6,6,5,5,6,5-ring system (**173**, **180**, and **181**) showed weak activities, with the exception of **171**, which showed potent inhibition. Analog **169** was the only one with a 6,6,6,5,6,5-ring system, and it was the most active among the analogs. Further investigation revealed that **169** significantly inhibited NLRP3 inflammasome activation and blocked NLRP3-inflammasome-induced pyroptosis via the amelioration of mitochondria damage, indicating that **169** can be used as a potential anti-inflammasome lead compound for further structure optimization [76]. Equisetin (**182**), a hemiterpene compound isolated from marine-sponge-derived fungi, displayed anti-atherosclerosis effects through inhibiting macrophage inflammatory response, lipid uptake, and foam cell formation in vitro, and finally ameliorated high-fat diet (HFD)-induced atherosclerosis in AopE-/- mice in vivo. Mechanistically, **182** directly bound to STAT3 with high affinity by forming hydrophobic bonds at GLN247 and GLN326 residues, as well as hydrogen bonds at ARG325 and THR346 residues, interacted with STAT3 physically, and functionally inhibited the transcription activity of STAT3, thereby regulating atherosclerosis. Therefore, these results indicated that **182** can be used as a candidate for developing anti-atherosclerosis therapeutic agents [77]. Four new indole diterpenoids, penpaxilloids A (**183**), C (**184**), and D (**185**) and 7-methoxypaxilline-13-ene (**186**), together with seven known analogues, schipenindolene A (**187**), 21-isopentenylpaxilline (**188**), penerpene E (**189**), paspalinine (**190**), 4a-demethylpaspaline-4a-carboxylic acid (**191**), paxilline D (**192**), and 7-methoxypaxilline (**193**), were isolated from the fungus, *Penicillium* sp. ZYX-Z-143, obtained from an arthropod, *Dardanus scutellatus*, collected from Yinyu Island in South China. Compounds **183**–**193** exhibited inhibitory activities toward NO production on LPS-stimulated RAW264.7 macrophages, with IC_50_ values of 33.09, 27.25, 7.11, 38.79, 11.87, 32.95, 23.89, 19.34, 28.22, 4.46, and 22.88 µM respectively, which were comparable to or better than those of the positive control (indomethacin, IC_50_ = 32.52 ± 2.90 μM). Additionally, compounds **185**, **187**, and **192** exhibited inhibitory activities toward NO production on LPS-stimulated RAW264.7 macrophages, displayed more potent anti-inflammatory activity than indomethacin, and showed no obvious cytotoxicity [78] (Figure 9).

**Figure 10:**
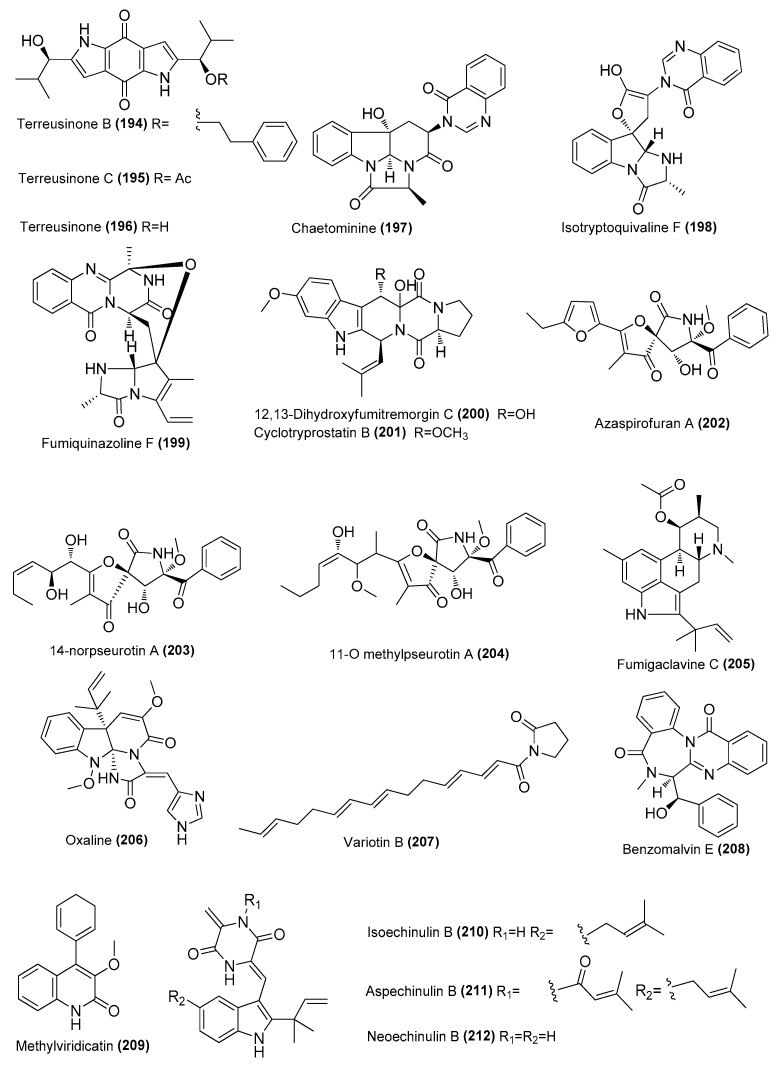

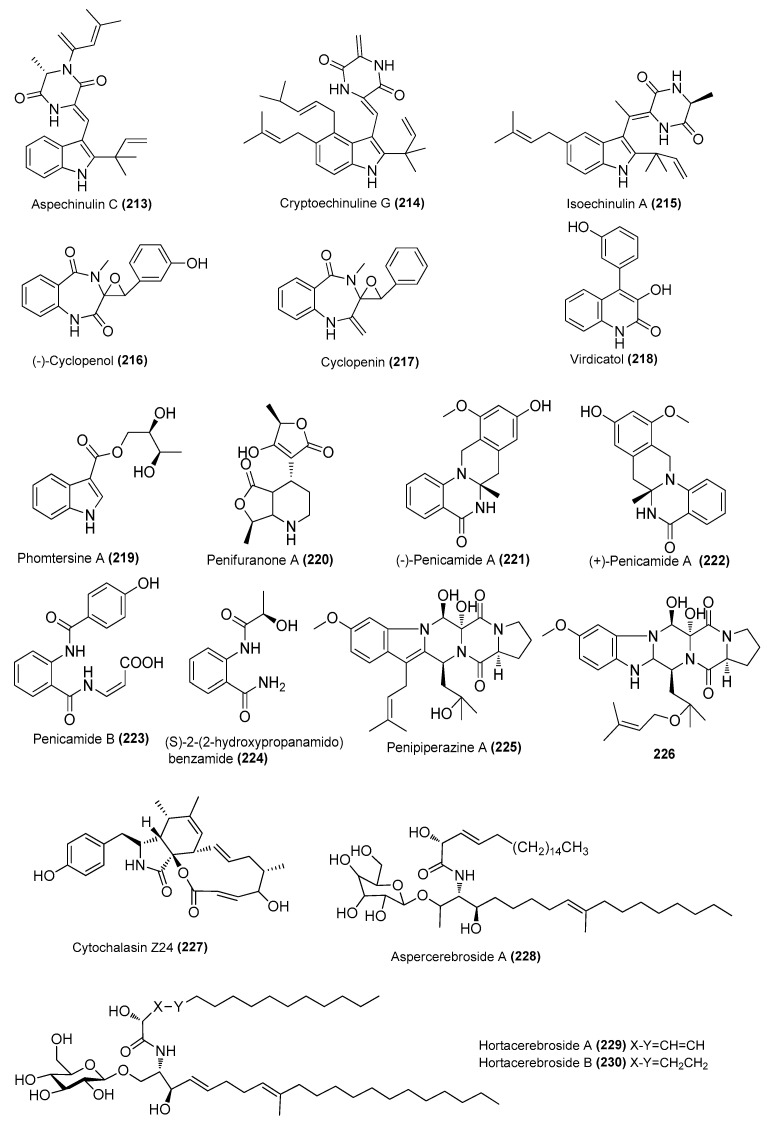
Alkaloids produced by marine fungi with anti-inflammatory activity (**194**−**230**).

**Scheme 4:**
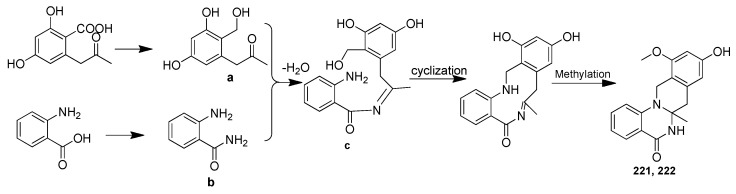
Postulation of the biogenetic relationships of **221**−**222** [86].

Two new dipyrrolobenzoquinones, terreusinones B (**194**) and C (**195**), along with the known analogue, terreusinone (**196**), were isolated from the marine-sponge-fibrosa-derived fungus, *Aspergillus tamarii* MCCF102 (Vizhinjam, Southwest coast of India). They exhibited NO-inhibitory activity in LPS-stimulated RAW 264.7 cells, with the IC_50_ values of 0.046, 0.096, and 0.032 µM, respectively [79]. Nine known alkaloids, chaetominine (**197**), isotryptoquivaline F (**198**), fumiquinazoline F (**199**), 12,13-dihydroxyfumitremorgin C (**200**), cyclotryprostatin B (**201**), azaspirofuran A (**202**), 14-norpseurotin A (**203**), 11-*O*-methylpseurotin A (**204**), and fumigaclavine C (**205**), were isolated from the South China Sea cold-seep-derived fungus. *Talaromyces helicus* SCSIO41311. Compound **202** showed more potent NO-inhibitory activities than EPA, with an IC_50_ value of 9.65 μM. Compounds **197** and **203** showed stronger NO-inhibitory activities than EPA, with IC_50_ values of 9.65 and 15.54 μM, respectively. Compounds **198**–**205** showed moderate inhibitory activities, with IC_50_ values of 26.51, 21.35, 24.95, 29.58, 32.37, 32.22, and 23.46 μM, respectively, while compound **197** exhibited weak inhibitory activities, with an IC_50_ value of 103.2 μM. Moreover, compound **203** could significantly attenuate the release of LPS-induced pro-inflammatory cytokines, such as TNF-*α* and INF-*γ*, while dramatically upregulating anti-inflammatory cytokines IL-4 and IL-10 [71]. One alkaloid, oxaline (**206**), was isolated from a fermented culture of the sponge-associated fungal strain, *Penicillium oxalicum* CLC-MF05 (Cu Lao Cham islands, Quang Nam, Vietnam). It inhibited the LPS-induced production of NO in BV-2 cells, with an IC_50_ value of 9.2 ± 0.5 μM, and showed inhibitory effects on the overproduction of NO and PGE2, the overexpression of iNOS and COX-2, and the mRNA overexpression of the pro-inflammatory cytokines, TNF-*α*, IL-1*β*, IL-6, and IL-12 in LPS-stimulated BV2 and rat primary microglia. The inhibitory effect of **206** was found to be regulated by the inactivation of the NF-*κ*B, MAPK, and TLR4/MyD88 signaling pathways, indicating that **206** presented potential anti-inflammatory candidates for the treatment of neurodegenerative diseases [58]. One new nitrogen-containing secondary metabolite, variotin B (**207**) was separated from the deep-sea fungus *Aspergillus unguis* IV17-109, based on NMR guided isolation. Compound **207** showed moderate anti-inflammatory activity, with an IC_50_ value of 20.0 µM [80]. Two compounds, benzomalvin E (**208**) and methylviridicatin **(209**), were isolated from the seawater-derived fungus, *Metarhizium* sp. P2100 (Qingdao Huiquan Bay, Yellow Sea, China), using the OSMAC strategy. Compounds **208** and **209** demonstrated anti-inflammatory activity against NO production induced by LPS, with IC_50_ values of 37.08 and 37.48 µM, respectively [81]. Two new diketopiperazine alkaloids, aspechinulins B (**211**) and C (**213**), along with four known ones, isoechinulin B (**210**), neoechinulin B (**212**), cryptoechinuline G (**214**), and isoechinulin A (**215**), were isolated from the deep-sea-derived fungus, *Aspergillus* sp. nFS445, in the Indian Ocean. Compounds **210**–**215** exhibited potential inhibitory activities against NO production in LPS-induced mouse marcrophage RAW 264.7, with IC_50_ values in the range of 20–90 µM, with compounds **210** and **213** shown to be as effective as the positive control, aminoguanidine (IC_50_, 23.7 µM) [82]. Three alkaloids, (-)-cyclopenol (**216**), cyclopenin (**217**), and virdicatol (**218**), were isolated from the marine-derived fungus, *Aspergillus austroafricanus* Y32-2, from the Indian Ocean. Compounds **216**–**218** displayed anti-inflammatory activity in a dose-dependent manner. Compound **218** displayed potent anti-inflammatory activity at a concentration of 30 µg/mL, and compounds **216** and **217** had moderate effects at concentrations of 70 and 120 µg/mL, respectively [83]. One new 3-carboxy-indole derivative, phomtersine A (**219**), was isolated from the marine deep-sea-derived fungus, *Phomopsis tersa* FS441, from the Indian Ocean. Its structure and absolute configuration were sufficiently established by spectroscopic methods and the modified Snatzke method. Compound **219** demonstrated anti-inflammatory activity against NO production induced by LPS, with an IC_50_ value of 83.57 ± 2.81 µM [84]. One new alkaloid, penifuranone A (**220**), isolated from the mangrove endophytic fungus, *Penicillium crustosum* SCNU-F0006, exhibited strong anti-inflammatory activity in vitro by inhibiting NO production in LPS-activated RAW264.7 cells, with an IC_50_ value of 42.2 µM. The docking study revealed that **220** exhibited an ideal fit within the active site of the murin iNOS, establishing characteristic hydrogen bonds [85]. An unreported N, N-ketal quinazolinone enantiomers (±)-penicamide A [(-)-**221** and (+)-**222**], and two known compounds, penicamide B (**223**) and (*S*)-2-(2-hydroxypropanamido) benzamide (**224**), were isolated from the ascidian *Styela plicata*-derived fungus, *Penicillium* sp. 4829 (Bay of Da’ao, Shenzhen City, Guangdong, China). The enantiomeric pair of (±)-penicamide A was the first example of a naturally occurring N,N-ketal quinazolinone possessing a unique tetracyclic system, having 4-quinazolinone fused with a tetrahydroisoquinoline moiety. (±)-Penicamide A should be PKS-NRPS hybrid metabolites derived from anthranilic acid and phenylpropanoid, 2,4-dihydroxy-6-(2-oxopropyl)benzoicacid. A possible biogenetic pathway for (±)-penicamide A was proposed, as shown in Scheme 4 [86]. Two intermediates, a and b, were derived from the precursors anthranilic acid and 2,4-dihydroxy-6-(2-oxopropyl)benzoic acid, followed by dehydration–condensation to generate an intermediate Schiff base, c. Subsequently, c underwent dehydration, cyclization, and methylation to give (±)-penicamide A. The enantiomeric mixtures of **221** and **222** displayed an inhibitory effect on NO production in LPS-activated RAW264.7 cells, while the optically pure (−)-**221** showed better inhibitory effects than (+)-**222**. The enantiomer mixture of (±)-penicamide A (**221** and **222**) displayed moderate inhibitory effects on NO production, with an IC_50_ value of 35.1 ± 1.7 µM, while the optically pure **221** showed better inhibitory effects than **222** (IC_50_: 27.2 ± 1.2 µM for **221** and 47.5 ± 2.3 µM for **222)**. In addition, **223** and **224** also exhibited moderate anti-inflammatory activity, with IC_50_ values of 45.9 ± 2.0 and 21.8 ± 1.3 µM, respectively [86]. Two novel diketopiperazine alkaloids, penipiperazine A (**225**) and its biogenetically related new metabolite (**226**), were obtained from the strain, *Penicillium brasilianum* HBU-136 (Bohai Sea, China; MH377073). Their planar structures and absolute configurations were elucidated by extensive spectroscopic analyses, ^13^C NMR calculation, and Marfey’s, ECD, and ORD methods. Compound **225** featured a unique 6/5/6/6/5 indole–pyrazino–pyrazino–pyrrolo system, and its plausible biogenetic pathway was also proposed, which was started from L-Pro and L-Trp, two important precursors to the synthesis of many 2,5-diketopiperazines in fungi [87]. Compounds **225** and **226** significantly inhibited the release of NO and the expression of related pro-inflammatory cytokines on LPS-stimulated RAW264.7 cells. They could markedly decrease the mRNA levels of pro-inflammatory cytokines, including IL-1*β*, IL-6, and TNF-*α*, in RAW264.7 cells stimulated by LPS, at a concentration of 25.0 µM, suggesting that they could be attractive candidates for further development as anti-inflammatory agents [87]. One known compound, cytochalasin Z24 (**227**), was isolated from marine-derived *Eutypella scoparia* GZU-4-19Y (Xuwen in Guangdong Province, China). Compound **227** showed potent NO inhibition activity, with an IC_50_ value of 17.1 μM [30]. A novel ceramide compound, aspercerebroside A (**228**), isolated from the EtOAc layer of the marine symbiotic fungus, *Aspergillus* sp. (Dongshan Island, Fujian Province, China), exhibited notable anti-inflammatory activity by effectively inhibiting the production of NO in RAW 264.7 cells at concentrations of 30 and 40 μg/mL, offering a promising avenue for the treatment of inflammatory diseases [88]. Two new cerebroside metabolites, hortacerebrosides A (**229**) and B (**230**), were isolated from the sponge-derived fungus, *Hortaea werneckii* (Danzhou, Hainan, China). Compounds **229** and **230** showed significant inhibitory effects on NO production by LPS-stimulated RAW 264.7 macrophages, with IC_50_ values of 7 and 5 μM, respectively, suggesting the potential application of these cerebrosides as drug leads targeting inflammation-related disorders [89] (Figure 10).

#### 2.3.2. Peptides

**Figure 11:**
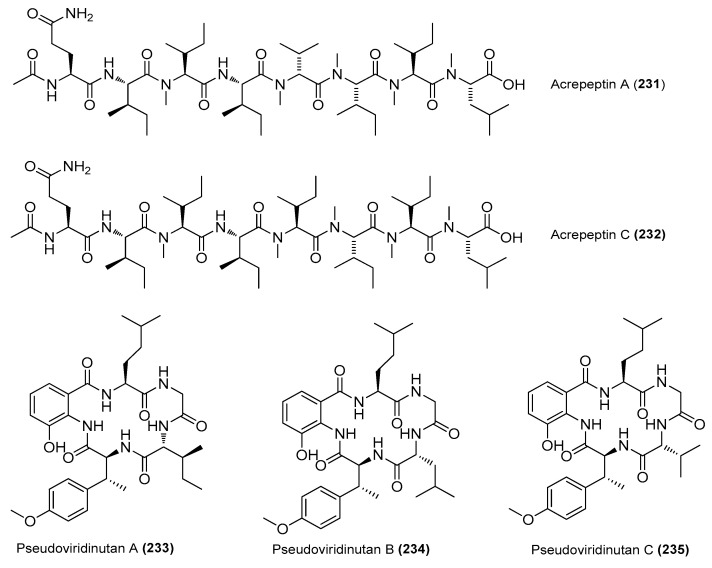

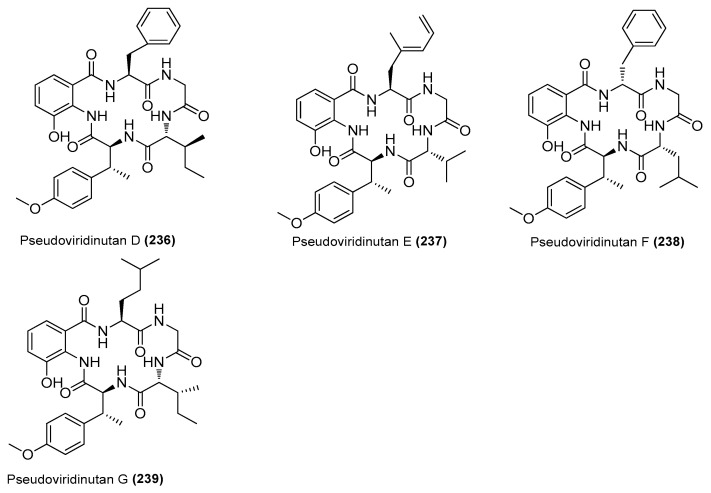
Peptides derived from marine fungi with anti-inflammatory activity (**231**−**239**).

Two new peptides, acrepeptins A (**231**) and C (**232**), were isolated from the red alga *Mastophora rosea-*derived fungal strain, *Acremonium* sp. NTU492, in the northeastern intertidal zone of Taiwan, and showed markedly inhibitory activities on nitric oxide production in LPS-activated microglial BV-2 cells, with IC_50_ values of 12.0 ± 2.3 and 10.6 ± 4.0 μM, respectively. Furthermore, they significantly attenuated the expression of inducible NO synthase in a concentration-dependent manner (5−40 μM) [90]. Seven new cyclopentapeptides, pseudoviridinutans A−G (**233**−**239**), were obtained from the ahydrothermal vent sediment-derived fungus, *Aspergillus pseudoviridinutans* TW58-5 (Kueishantao, Taiwan, China), by a molecular-networking-guided isolation procedure. Those compounds feature a rare amino acid moiety, *O*,*β*-dimethyltyrosine, observed for the first time in a marine-derived fungus, and their absolute configurations were determined using a combination of Marfey’s method and X-ray diffraction. Compounds **233**−**239** showed anti-inflammatory effects on the production of NO stimulated by LPS on cultured RAW264.7 cells, especially **239**, which displayed obvious inhibitory effects at 20 μM, with no obvious cytotoxicity. Compound **239** inhibited NO production in LPS-induced murine macrophage RAW264.7 cells by regulating the expression levels of NLRP3 and iNOS [91] (Figure 11).

### 2.4. Steroids

**Figure 12:**
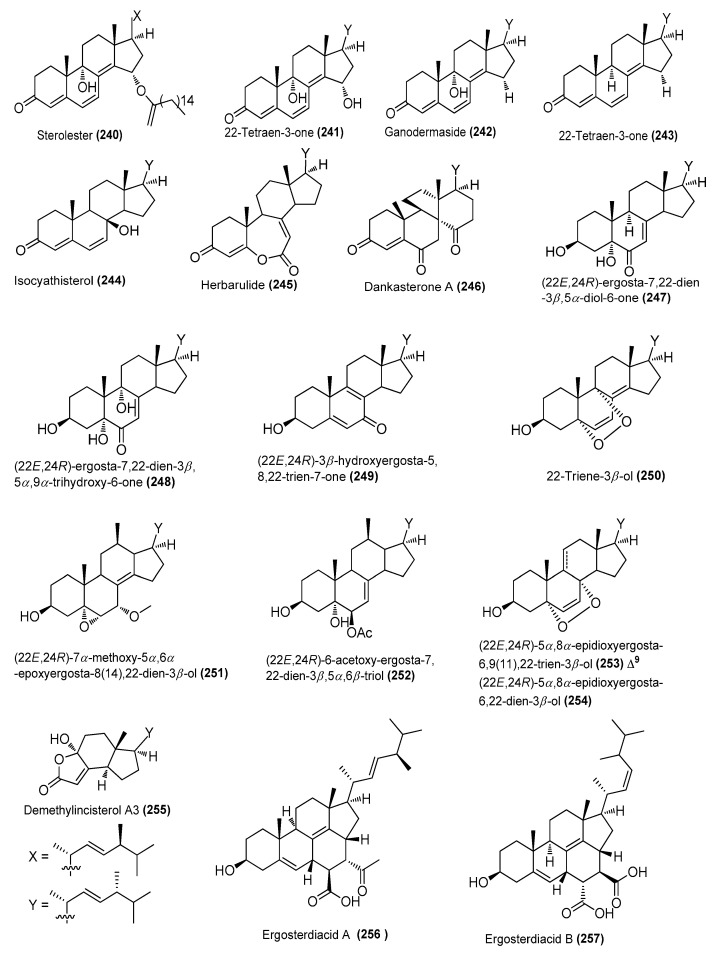

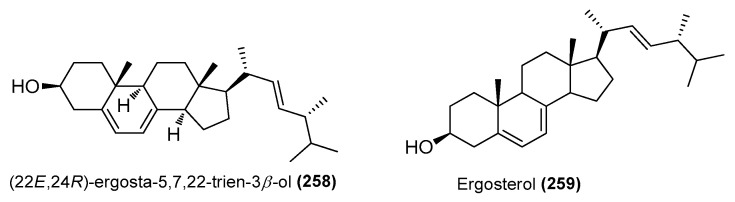
Steroids produced by marine-derived fungus with anti-inflammatory activity (**240**−**259**).

Steroids are biosynthesized via intricate cyclization reactions that involve the squalene and mevalonate pathways. Twenty anti-inflammatory steroids have been identified from marine-derived fungi. Among them, three are novel compounds, and two of these new compounds (**256** and **257**) possess novel skeletal structures (Figure 12).

One new ergostane-type sterolester (**240**), along with 15 known compounds, 22-tetraen-3-one (**241**), ganodermaside (**242**), 22-tetraen-3-one (**243**), isocyathisterol (**244**), herbarulide (**245**), dankasterone A (**246**), (22*E*,24*R*)-ergosta-7,22-dien-3*β*,5*α*-diol-6-one (**247**), (22*E*,24*R*)-ergosta-7,22-dien-3*β*,5*α*,9*α*-trihydroxy-6-one (**248**), (22*E*,24*R*)-3*β*-hydroxyergosta-5,8,22-trien-7-one (**249**), 22-triene-3*β*-ol (**250**), (22*E*, 24*R*)-7*α*-methoxy-5*α*,6*α*-epoxyergosta-8(14),22-dien-3*β*-ol (**251**), (22*E*,24*R*)-6-acetoxy-ergosta-7,22-dien-3*β*,5*α*,6*β*-triol (**252**), (22*E*,24*R*)-5*α*,8*α*-epidioxyergosta-6,9(11),22-trien-3*β*-ol (**253**), (22*E*,24*R*)-5*α*,8*α*-epidioxyergosta-6,22-dien-3*β*-ol (**254**), and demethylincisterol A3 (**255**), were isolated from the fungus, *Penicillium oxalicum* HL-44, associated with the soft coral, *Sinularia gaweli* (Xisha area of the South China Sea). These compounds demonstrated potent anti-inflammatory activities at a concentration of 20 µM. Compounds **241**, **248**, and **253** exhibited significant inhibition of IFNB1 expression, while compounds **242**, **243**, and **244** showed strong inhibition of TNF-*α* expression in LPS-stimulated cells. In DT-DIAPHORASE inhibitor (DMXAA)-stimulated cells, compounds **240**, **244**, and **246** effectively suppressed IFNB1 expression, whereas compounds **246**, **247**, and **250** demonstrated the most potent inhibition of TNF-*α* expression. These findings suggest that these tested compounds may exert their anti-inflammatory effects by modulating the cGAS-STING pathway. This study provided valuable insight into the chemical diversity of ergosteroid derivatives and their potential as anti-inflammatory agents [92]. Two unusual naturally Diels–Alder additive steroids, ergosterdiacids A and B (**256** and **257**), constructing a 6/6/6/6/5 pentacyclic steroidal system, were obtained from the mangrove plant *Aegiceras corniculatum*-derived fungus *Aspergillus* sp. (Thailand). The plausible biosynthetic pathways of **256** and **257** were discussed. Compounds **256** and **257** should be naturally Diels–Alder addition products between fumaric acid and ergosta-5,7,14,22-tetraene-3*β*-ol. They were derived from the precursor steroids, (22*E*,24*R*)-ergosta-5,7,14,22-tetraene-3*β*-ol and (22*Z*,24*ξ*)-ergosta-5,7,14,22-tetraene-3*β*-ol, respectively, which were probably generated with fumaric acid by an enzymatically catalyzed reaction [37]. Moreover, **256** and **257** showed strong in vitro anti-inflammatory effects by suppressing NO production at 4.5 and 3.6 μM, respectively [93]. One known compound, (22*E*,24*R*)-ergosta-5,7,22-trien-3*β-*ol **(258**), was isolated from the mangrove *Avicennia marina* endophytic fungus, *Amorosia* sp. SCSIO 4102 (Zhanjiang, Guangdong Province, China). It could inhibit the LPS-induced overproduction of NO and pro-inflammatory cytokines, including IL-6, TNF-*α*, and MCP-1, in both mRNA and protein levels with the condition that none of these compounds showed significant cytotoxicity [36]. One known compound, ergosterol (**259**), was obtained from the deep-sea sulfide-sample-derived fungus, *Samsoniella hepiali* W7 (South Atlantic), by using the molecular networking approach. Under a concentration of 1 µM, compound **259** showed potent inhibitory activity against NO production in LPS-activated BV-2 microglia cells, with an inhibition rate of 32.9% [46] (Figure 12).

### 2.5. Other Classes

Additionally, there are also 26 other classes of anti-inflammatory secondary metabolites (including 14 new compounds) isolated from marine-derived fungi, including fatty acids and benzene derivatives.

**Figure 13:**
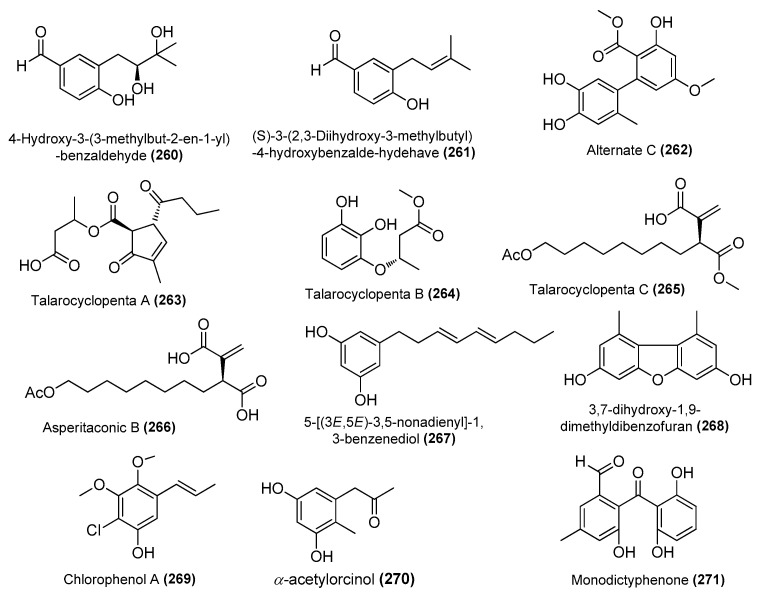

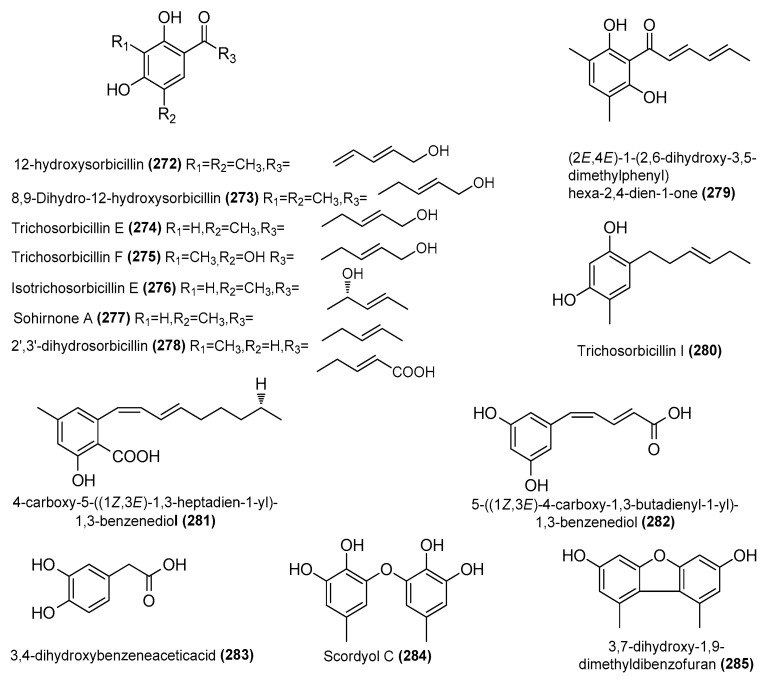
Other classes produced by marine fungi with anti-inflammatory activity (**260**−**285**).

One new benzaldehyde, 4-hydroxy-3-(3-methylbut-2-en-1-yl)-benzaldehyde (**261**), and one known compound, (*S*)-3-(2,3-dihydroxy-3-methylbutyl)-4-hydroxybenzalde-hydehave (**260**), were isolated from a coral-derived *A. terreus* strain, C23-3. Compounds **260** and **261** showed anti-inflammatory effects via the suppression of the MAPK signaling pathway in RAW264.7 cells. They could reduce the levels of some inflammatory biomarkers, significantly inhibit the release of NO and ROS, and effectively block the protein expression of IL-6, iNOS, and COX-2 and the phosphorylation levels of ERK, JNK, and p38 [94]. One known secondary metabolite alternate C (**262**) was isolated from the Antarctic fungal strain, *Pleosporales* sp. SF-7343 (King George Island, Antarctica), and it inhibited the secretion of IL-8 and IL-6 in tumor necrosis factor-*α*/interferon-*γ*-treated HaCaT cells in an inflammatory disease, atopic dermatitis [47]. One novel cyclopentenone derivative, talarocyclopenta A (**263**), one new phenolic derivative, talarocyclopenta B (**264**), and one new itaconic acid derivative, talarocyclopenta C (**265**), together with one known itaconic acid derivative, asperitaconic B (**266**), were isolated from the leaves of the *Ceriops tagal* fungus, *Talaromyces assiutensis* JTY2, from the South China Sea. Compounds **263**−**266** showed significant anti-inflammatory activities against NO production induced by LPS in mouse macrophage RAW 264.7 cells in vitro, with IC_50_ values of 3.38 ± 0.12, 6.26 ± 0.10, 12.56 ± 0.08, and 15.63 ± 0.12 μM, respectively, while the positive control, hydrocortisone, showed inhibitory activity, with an IC_50_ value of 3.68 ± 0.10 μM [95]. One new compound, 5-[(3*E*,5*E*)-3,5-nonadienyl]-1,3-benzenediol (**267**), was isolated from a marine brown alga *Saccharina cichorioides*-derived *Aspergillus* sp., from the South China Sea. Compound **267** significantly inhibited NO production, with an IC_50_ value of 6.0 ± 0.5 μM, in LPS-induced RAW264.7 cells. Moreover, compound **267** also showed anti-inflammatory activity by inhibiting the NF-κB activated pathway [96]. One known compound, 3,7-dihydroxy-1,9-dimethyldibenzofuran (**268**), was isolated from the deep-sea-sediment-derived fungus, *Aspergillus sydowii* MCCC 3A00324 (South Atlantic Ocean). Compound **268** showed potent inhibitory NO production in LPS-activated BV-2 microglia cells, with an inhibition rate of 94.4%, at a concentration of 10 µM [24]. One new compound, chlorophenol A (**269**), and one known compound, *α*-acetylorcinol (**270**), were isolated from the mangrove *Avicennia marina* endophytic fungus, *Amorosia* sp. SCSIO 4102 (Zhanjiang, Guangdong Province, China). Compounds **269** and **270** inhibited LPS-induced overproduction of NO and pro-inflammatory cytokines, including IL-6, TNF-*α*, and MCP-1, in both mRNA and protein levels, with the condition that none of these compounds showed significant cytotoxicity [36]. One compound, monodictyphenone (**271**), isolated from the ascidian-derived fungus, *Diaporthe* sp. SYSU-MS4722, showed anti-inflammatory activity, with an IC_50_ value of 40.8 µM (positive control indomethacin, IC_50_ = 35.8 µM) [38]. Six new monomeric sorbicillinoids, 12-hydroxysorbicillin (**272**), 8,9-dihydro-12-hydroxysorbicillin (**273**), trichosorbicillin E (**274**), trichosorbicillin F (**275**), isotrichosorbicillin E (**276**), and trichosorbicillin I (**280**), along with three known compounds, sohirnone A (**277**), 2′,3′-dihydrosorbicillin (**278**), and (2*E*,4*E*)-1-(2,6-dihydroxy-3,5-dimethylphenyl)hexa-2,4-dien-1-one (**279**), were isolated from the marine-sponge-derived fungus, *Trichoderma reesei* 4670 (Shantou, Guangdong Province, China). Compounds **272**−**280** exhibited potent anti-inflammatory activity by inhibiting the production of NO in RAW264.7 cells activated by LPS, with IC_50_ values of 6.8, 2.9, 0.94, 6.1, 12, 14, 13, 3.3, and 13 μM, respectively. A preliminary SAR analysis indicated that the anti-inflammatory activities of the sorbicillinoids mainly depended on the structural types and the functional groups of the sorbyl side chain. For monomeric sorbicillinoids, the double bonds of the sorbyl side chain played an important role in their anti-inflammatory action, as compounds **272**−**280**, with at least one double bond in the side chain, were much more active than the compounds without a double bond in the side chain. Compounds **274** and **277** exhibited stronger anti-inflammatory effects, indicating that the terminal carboxylic acid group of the sorbyl side chain was a disadvantage for anti-inflammatory activity. The keto carbonyl group at C-7 made no difference to the anti-inflammatory activity. Compound **277**, with a keto carbonyl group at C-7, showed the same level of activity as **280**, which contained a methylene group at C-7. In the case of dimeric sorbicillinoids, the presence of a lipophilic terminus on the sorbyl side chain appeared to enhance anti-inflammatory activity better than when a hydrophilic group was presented [64]. Two new compounds, 4-carboxy-5-((1*Z*,3*E*)-1,3-heptadien-1-yl)-1,3-benzenediol (**281**) and 5-((1*Z*,3*E*)-4-carboxy-1,3-butadienyl-1-yl)-1,3-benzenediol (**282**), along with one known compound, 3,4-dihydroxybenzeneaceticacid (**283**), were isolated from the deep sea hydrothermal vent sediment-derived fungus *Penicillium* sp. TW58-16 (Kueishantao, Taiwan). Compounds **281**−**283** suppressed LPS-stimulated NO production in macrophages, and, in particular, **281** greatly inhibited the expression of iNOS, the enzyme that produces NO [52]. Two known compounds, scordyol C (**284**) and 3,7-dihydroxy-1,9-dimethyldibenzofuran (**285**), were isolated from a strain of the sponge-derived marine fungus, *Aspergillus carneus* GXIMD00543 (Weizhou islands coral reef, Beibu Gulf, China). Compounds **284** and **285** exhibited moderate and potent anti-inflammatory effects on NO secretion when using LPS-induced RAW 264.7 cells, with EC_50_ values of 26.8 ± 1.7 and 2.9 ± 0.1 μM, respectively [72] (Figure 13).

## 3. Conclusions

This review provides a summary of the sources, structural diversity, and biological activities of secondary metabolites produced by marine fungi, encompassing a time frame from August 2018 to July 2024. A total of 285 anti-inflammatory compounds were isolated from marine-derived fungi, and 156 were new compounds. Due to the high salt levels of the marine environment, some nitrogen-containing secondary metabolites from marine-derived fungi contained halogen atoms, such as compounds **33**, **37**, and **259**–**262**. Remarkably, among them, 11 compounds (**1**, **46**, **64**, **136**−**139**, **221**, **222**, **256,** and **257**) exhibited novel skeletal structures, and the proposed biosynthetic pathways of novel skeleton structures were also discussed. The isolated compounds, along with their biological activities, producing strains, and habitats, are summarized in Table 1.

As shown Table 1, about 47 compounds displayed significantly anti-inflammatory activities comparable to or better than the positive control. Examples of these include paraconulones B−E (**3**−**6**), paraconulone G (**8**), 4-epi-microsphaeropsisin (**11**), eremofortin H (**15**), nectriapyrone (**62**), sequoiatone B (**149**), diaporspchromanone B**,** oxaline (**206**), and isoechinulin B (**210**). The preliminary SAR values of the bioactive compounds were also discussed. The mechanisms of 46 compounds with potent inflammatory activity, such as sesquiterpene (**22**), aspergillon A (**39**), peniscmeroterpenoid D (**45**), butyrolactone I (**78**), ravenelin (**112**), and sclerotiamide J (**226**), were also demonstrated. The preliminary SAR of the isolated isopimarane-type diterpenoids (**23**−**38**), polyketides (**56**−**59)**, sorbicillinoids (**120**−**128**), and notoamidetype alkaloids (**173**−**185** and **276**−**284**) were also discussed. The inhibitory effects of the above compounds are regulated by the inactivation of the NF-*κ*B, MAPK, and TLR4/MyD88 signaling pathways. Furthermore, various proteins associated with inflammation were examined within the aforementioned signaling pathways in immune monocytes and macrophages (BV2 cells and RAW264.7 cells), stimulated by LPS in vitro. The impact on the swelling rate was assessed using a mouse ear edema model induced by phorbol myristate acetate in vivo. Furthermore, 15 compounds (**22**, **26**, **41**, **58**, **71**, **78**, **80**, **136**, **152**, **157**, **158**, **163**, **169**, **182**, and **206**), exhibiting potent anti-inflammatory activity, have the potential to serve as anti-inflammatory candidates.

**Figure 14:**
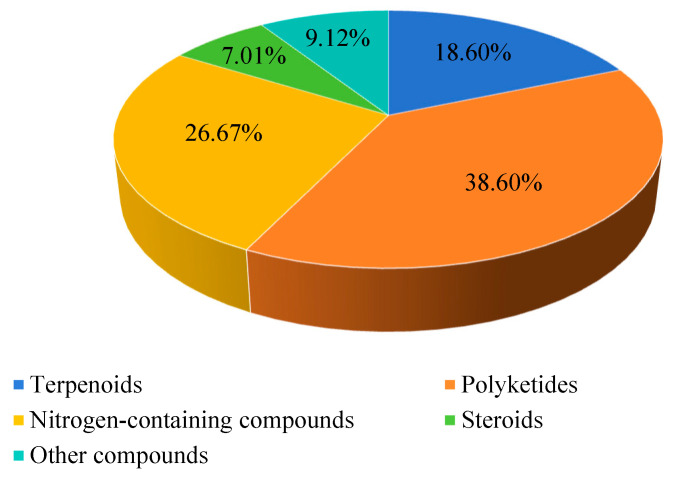
The numbers and types of anti-inflammatory compounds isolated from marine fungi.

The distribution of the structural types and bioactivity among anti-inflammatory compounds derived from marine fungi is also depicted in Figure 14. The chemical structures of the 285 secondary metabolites from marine-derived fungi were mainly classified into five types, including 53 terpenoids, 110 polyketides, 76 nitrogen-containing compounds, 20 steroids, and 26 other compounds. Among these compounds, polyketides accounted for the largest proportion, at 38.60%, followed by nitrogen-containing compounds accounted, with 26.67%. Terpenoids accounted for 18.60%, and steroids and other classes accounted for 7.01%, and 9.12%, respectively (Figure 14). From a distributional perspective, 65.27% of all anti-inflammatory structures were polyketides (38.60%) and nitrogen-containing compounds (26.67%), indicating that polyketides and nitrogen-containing compounds have great potential in the development of anti-inflammatory drugs.

**Figure 15:**
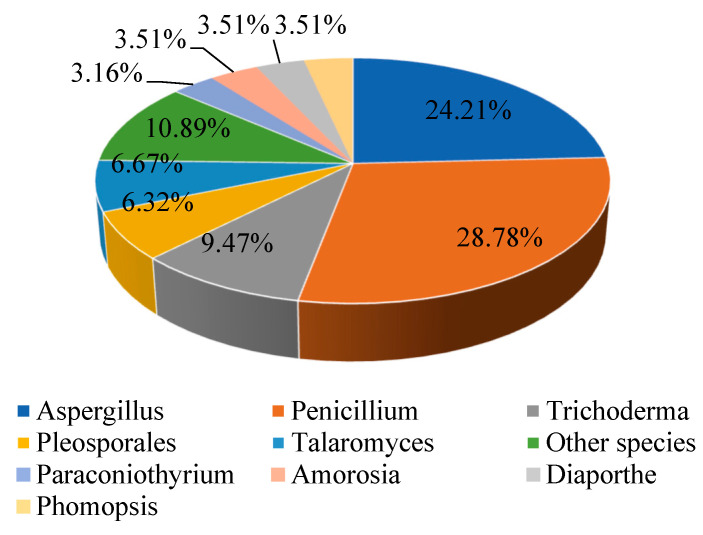
The proportion of anti-inflammatory compounds from different marine fungi sources.

**Figure 16:**
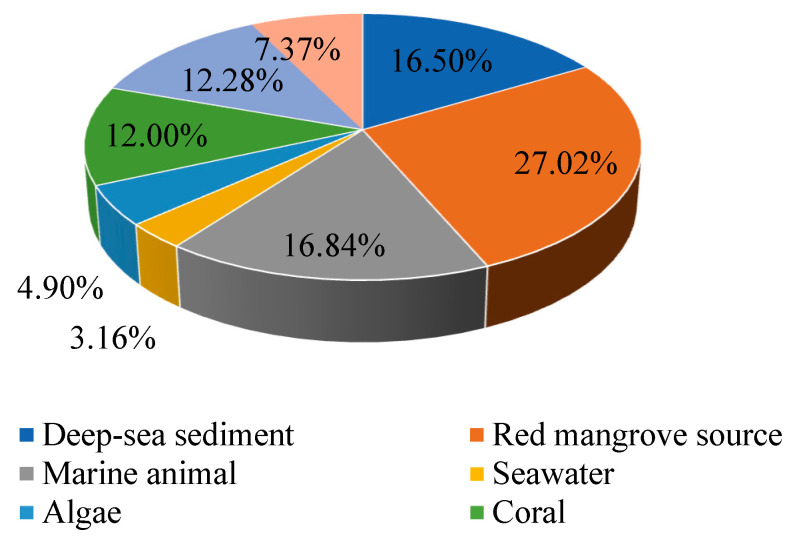
The proportion of marine-derived fungi from different marine sources.

This review has identified numerous potential lead compounds that could lead to the discovery of innovative anti-inflammatory agents originating from fungi sourced from marine environments., especially *Aspergillus* sp. (41.4%) and *Penicillium* sp. (27.1%) (Figure 15). Additionally, the samples were collected from various environments: 16.50% from sediment, 12.0% from corals, 12.28% from sponges, 27.02% from mangroves, 3.16% from seawater, and 16.84% from marine animals, 4.9% from algae, and 7.37% from other marine resources (Figure 16).

In summary, marine-derived fungi were proven to be important sources of novel structures and diverse secondary metabolites with anti-inflammatory activities, revealing their great untapped potential in medicinal applications. Marine-derived fungi hold promise as sources for bioprospecting safe and effective anti-inflammatory agents to tackle these curable, yet potentially devastating conditions. The purpose of this review is to offer insights into the advancement of research and to furnish additional momentum for the transformation of compounds with distinctive structural features derived from marine fungi into anti-inflammatory medications. However, despite the promising anti-inflammatory significance of marine-fungi-derived compounds and extracts, there are still no FDA-approved marine-fungi-derived anti-inflammatory drugs. The transfer of technology from experimental outcomes to pre-clinical and clinical applications of secondary metabolites derived from marine fungi remains in its nascent stages and has not yet fully harnessed the pharmaceutical potential of these compounds. In the future, our focus should be on elucidating the pharmacological mechanisms, understanding the pharmacokinetics, advancing medicinal chemistry, and exploring biosynthesis to foster the development of innovative drugs in subsequent research.

## Figures and Tables

**Table 1:** The biological activities, producing strains, and habitats of anti-Inflammatory secondary metabolites from marine-derived fungi.

Compounds	Producing Strains	Habitats	Genbank Accession Number	Bioactivities	References
Aspermonoterpenoid A (**1**)	*Aspergillus sydowii* MCCC 3A00324	Deep-sea sediment, South Atlantic Ocean	MN918102	Inhibited NO production in LPS-induced RAW 264.7 cells at 20 µM	[24]
Aspermonoterpenoid B (**2**)	*A. sydowii* MCCC 3A00324	Deep-sea sediment, South Atlantic Ocean	MN918102	Inhibited NO production in LPS-induced RAW 264.7 cells at 10 µM	[24]
Paraconulones B−E (**3**−**6**)	*Paraconiothyrium sporulosum* DL-16	Coastal sediment, Bohai Bay, Liaoning, China	MZ505391	Inhibited NO production in LPS-induced RAW 264.7 cells, IC_50_ = 6.9 ± 2.6, 7.7 ± 2.0, 2.8 ± 0.5, 8.1 ± 2.9 μΜ, respectively	[25]
Paraconulone F (**7**)	*P. sporulosum* DL-16	Coastal sediment, Bohai Bay, Liaoning, China	MZ505391	Inhibited NO production in LPS-induced RAW 264.7 cells, IC_50_ = 40 ± 15 μΜ	[25]
Paraconulone G (**8**)	*P. sporulosum* DL-16	Coastal sediment, Bohai Bay, Liaoning, China	MZ505391	Inhibited NO production in LPS-induced BV2 cells, IC_50_ = 8.1 ± 3.5 μΜ	[25]
Periconianone D (**9**)	*P. sporulosum* DL-16	Coastal sediment, Bohai Bay, Liaoning, China Coastal sediment, Bohai Bay, Liaoning, China	MZ505391	Inhibited NO production in LPS-induced BV2 cells, IC_50_ = 98 ± 17 μΜ	[25]
Microsphaeropsisin (**10**)	*P. sporulosum* DL-16	Coastal sediment, Bohai Bay, Liaoning, China	MZ505391	Inhibited NO production in LPS-induced BV2 cells, IC_50_ = 80 ± 38 μΜ	[25]
4-epi-microsphaeropsisin (**11**)	*P. sporulosum* DL-16	Coastal sediment, Bohai Bay, Liaoning, China	MZ505391	Inhibited NO production in LPS-induced BV2 cells, IC_50_ = 4.6 ± 3.5 μΜ	[25]
AA03390 (**12**)	*Phomopsis* sp. SYSU-QYP-23	Mangrove, East Harbour National Nature Reserve, Hainan, China	MN871866	Inhibited NO production in LPS-induced RAW 264.7 cells, IC_50_ = 14.5 μΜ	[26]
Eremofortins G-J (**13-17)**	*Phomopsis* sp. SYSU-QYP-23	Mangrove, East Harbour National Nature Reserve, Hainan, China	MN871866	Inhibited NO production in LPS-induced RAW 264.7 cells, IC_50_ = 8.6−13.5 μΜ	[26]
lithocarin A (**18**)	*Phomopsis* sp. SYSU-QYP-23	Mangrove, East Harbour National Nature Reserve, Hainan, China	MN871866	Inhibited NO production in LPS-induced RAW 264.7 cells, IC_50_ = 10.5 μΜ	[26]
Ustusolates H-J (**19-20**)	*Aspergillus insuetus* SYSU6925	Seagrass, Zhuhai, Guangdong, China	MZ411391	Inhibited NO production in LPS-induced RAW 264.7 cells, IC_50_ = 21.5 and 32.6 μΜ, respectively	[27]
**21** and **22**	*Eutypella* sp. D-1	London Island, Arctic	FJ430580	Modulated the MAPK and NLRP3/caspase-1 signaling pathways	[28]
Pleosmaranes A-D (**23-26),** F (**27**), H–J (**28**–**30**), L (**31**), M (**32**), and O–R (**33**–**36**); sphaeropsidin C (**37**), pleosmarane S (**38**)	*Pleosporales* sp. HNQQJ-1	Mangrove, Dongzhai Harbor Mangrove Nature Reserve, Hainan, China	OR616722	Inhibited NO production in LP S induced RAW 264.7 cells, IC_50_ = 30, 37, 38, 42, 42, 19, 35, 33, 25, 35, 37, 30, 33, 35, 31 and 40 μM, compared with the positive control (L-NMMA, 33 μM).	[29]
Aspergillon A (**39**)	*Eutypella scoparia* GZU-4-19Y	Xuwen, Guangdong, China	OM920979	Inhibited NO production, IC_50_ = 2.0 μM, suppressed the protein expression of iNOS and COX-2 at 2.5 μM	[30]
Talaroacids A (**40**) and D (**42**), Talaromarane A (**43**)	*Talaromyces* sp. JNQQJ-4	Mangrove, Jinniu Island Mangrove Nature Reserve, Guangzhou, China	MK450749.1	Inhibited NO production, IC_50_ = 15.78, 21.60, and 13.38 μM, respectively	[31]
Talaroacid B (**41**)	*Talaromyces* sp. JNQQJ-4	Jinniu Island Mangrove Nature Reserve, Guangzhou, China	MK450749.1	Inhibited NO production, IC_50_ = 21.60 μΜ, positive control quercetin (IC_50_, 11.33 μM)	[31]
Peniscmeroterpenoids A and D (**44** and **45**)	*Penicillium sclerotiorum* GZU-XW03-2.	*Onchidium* sp., Guangdong, China.	MT071304)	Inhibited NO production, IC_50_ = 26.60 and 8.79 μM, respectively	[32]
Aspermeroterpene A-C (**46–48**)	*Aspergillus terreus* GZU 31-1	Guangdong province (Zhanjiang, Xuwen), China	MN860009	Inhibited NO production, IC_50_ (anti-inflammatory)17.8,14.1 and 13.4 μM	[33]
Terretonin D1(**49**), Terretonin (**50**)**,** Terretonins A and D (**51** and **52**)	*Aspergillus terreus* ML-44	*Pacific oyster*, Yangma Island in Yantai, China	CGMCC 15664	Inhibited NO production, inhibitory rates of 30.2%, 34.0%, 22.5% and 23.5%, at 50 μg/mL	[34]
Aspergienyne C (**53**)	*Aspergillus* sp. GXNU-Y65	Mangrove *Kandelia cande*, Beihai, China	MT626087	Aspergienyne C had strong anti-nonalcoholic steatohepatitis activity against AML12 cells treated with PA (palmitic acid) + OA (oleic acid).	[35]
Asperphenylpyrone (**54**)	*Aspergillus sydowii* MCCC 3A00324	Deep-sea sediment, South Atlantic Ocean	MN918102	Inhibited NO production in LPS-induced RAW 264.7 cells at 20 µM	[24]
Aspercoumarine acid (**55**)	*A. sydowii* MCCC 3A00324	Deep-sea sediment, South Atlantic Ocean	MN918102	Inhibited NO production in LPS-induced RAW 264.7 cells at 10 µM	[24]
6,8-dihydroxy-3-methylisocoumarine (**56**)	*Amorosia* sp. SCSIO 4102	Mangrove *Avicennia marina*, Zhanjiang, Guangdong, China	OL826791	Inhibited the production of inflammatory factors in both mRNA and protein levels	[36]
(S)-5,7-dichloro-6-methoxy-2-methyl-2,3-dihydrobenzofuran-4-carboxylic acid (**57**)	*Amorosia* sp. SCSIO 4102	Mangrove *Avicennia marina*, Zhanjiang, Guangdong, China	OL826791	Inhibited the production of inflammatory factors in both mRNA and protein levels	[36]
5-chloro-6-hydroxymellein (**58**)	*Amorosia* sp. SCSIO 4102	Mangrove *Avicennia marina*, Zhanjiang, Guangdong, China	OL826791	Inhibited the production of inflammatory factors in both mRNA and protein levels	[36]
3-methyl-6-hydroxy-8-methoxy-3,4-dihydroisocoumarin **(59**)	*Amorosia* sp. SCSIO 4102	Mangrove *Avicennia marina*, Zhanjiang, Guangdong, China	OL826791	Inhibited the production of inflammatory factors in both mRNA and protein levels	[36]
Amphichopyrones A (**60**) and B (**61**)	*Amphichorda felina* SYSU-MS7908	*Culturing ascidian*, Xisha Islands, South China Sea, China.	MT786206	Inhibited NO production in LPS-induced RAW 264.7 cells, IC_50_ = 18.09 and 7.18 μΜ, respectively	[37]
Nectriapyrone **(62)**	*Diaporthe* sp. SYSU-MS4722	Shenzhen City, Guangdong, Province, China	OK623372	Inhibited NO production in LPS-induced RAW 264.7 cells, IC_50_ = 35.4 μΜ, positive control indomethacin, IC_50_ = 35.8 µM	[38]
Trichopyrone (**63**)	*Penicillium* sp. DM815	Qinglan, Wenchang, Hainan Province	MW497629	Weakly inhibited LPS-induced NO release at 10 μM	[39]
Penicillol B (**64**)	*Penicillium* sp. BJR-P2	Mangrove *Avicennia marinav*, Yangjiang Hailing Island Mangrove Wetland Park, China	PRJNA793386	Inhibited NO production in LPS-induced RAW 264.7 cells, IC_50_ = 12 μΜ	[40]
Decempyrones C (**65**) and J (**66**)	*Fusarium decemcellulare* SYSU-MS6716	Sea grass, Lingshui Xincungang and Li’angang Special Protected Area, Hainan, China	MW851212	Inhibited NO production in LPS-induced RAW 264.7 cells, IC_50_ = 22.4 and 21.7 μΜ, respectively	[41]
5,9-dihydroxy-2,4,6,8,10-pentamethyldodeca-2,6,10-trienal (**67**)	*A. ochraceopetaliformis* SCSIO 41020	*Hypnea pannosa*, Sanya city, Hainan province, China	OL884728	Blocked the release of pro-inflammatory cytokines (IL-6, MCP-1, and TNF-*α*) induced by LPS both in vivo and in vitro	[42]
(3R,4*S*)-(−)-4-hydroxymellein (**68**)	*A. ochraceopetaliformi*s SCSIO 41020	*Hypnea pannosa*, Sanya city, Hainan province, China	OL884728	Inhibited NO production in LPS-induced RAW 264.7 cells	[42]
(3*R*,4*R*)-(−)-4-hydroxymellein (**69**)	*A.ochraceopetaliformis* SCSIO 41020	*Hypnea pannosa*, Sanya city, Hainan province, China	OL884728	Inhibited NO production in LPS-induced RAW 264.7 cells	[42]
sclerketide D (**70**)	*Penicillium sclerotiorum* CHNSCLM-0013	Gorgonian, Weizhou coral reef, South China Sea	KT695601	Inhibited NO production in LPS-induced RAW 264.7 cells, IC_50_ = 5.5 μΜ	[43]
Dicitrinone G (**71**)	*Penicillium* sp. GGF 16-1-2	in the South China Sea	_	Regulated the activation of NLRP3 infammasome	[44]
(4*S*,5*S*,6*S*,7*R*)-4-(3-chloro-1,2-dihydroxybutyl)-butyrolactone (**72**)	*Neofusicoccum parvum* Y2NBKZG1016	Mangrove *Sonneratia glauca*, Nansha District, Guangzhou, China	_	Weak anti-inflammatory activity at concentrations ≥ 6.25 μM	[45]
Walterolactone A (**73**)	*Samsoniella hepiali* W7	Deep-sea sulfide sample, South Atlantic	NR_160318.1	Inhibited NO production in LPS-activated BV-2 microglia cells, with inhibition rates of 38.6% at 1 µM	[46]
(4R,5S)-5-hydroxyhexan-4-olide (**74**)	*Samsoniella hepiali* W7	Deep-sea sulfide sample, South Atlantic	NR_160318.1	Inhibited NO production in LPS-activated BV-2 microglia cells, IC_50_ = 426.2 nM	[46]
Alternariol (**75**)	*Pleosporales* sp. SF-7343	King George Island, Antarctica	MK785420	Inhibited inflammatory factors	[47]
Aspulvinone U (**76**)	*Aspergillus terreus* NTU243	Marine alga *Ulva lactuca*, northeastern coast, Taiwan, China	PRJNA611016	Inhibited LPS-induced MMP-9-mediated gelatinolysis, inhibition rate of 56.0% at 10 µM	[48]
Aspulvinone V (**77**)	*A. terreus* NTU243	Marine alga *Ulva lactuca*, northeastern coast, Taiwan, China	PRJNA611016	Inhibited NO production in LPS-induced RAW 264.7 cells, and LPS-induced MMP-9-mediated gelatinolysis, with inhibition rates of 45.0% and 67.8%, 10 µM	[48]
Butyrolactone I (**78**)	*Aspergillus flavipes* MTCC 5220	Mangrove plant *Acanthus illicifolius*, Goa, India	PRJNA611016	IC_50_ (anti-inflammatory) 12.03 µM (IL-6), 43.29 µM (TNF-α)	[49]
Butyrolactone I (**78**)	*Aspergillus terreus* XWC21-10	Coral *Porites pukoensis*, Zhanjiang seawaters of the South China Sea	PRJNA611016	Inhibited the expression of iNOS and COX-2	[50]
Butyrolactone I (**78**)	*A. terreus var. africanus* IFO 8835	_	_	Regulating inflammation by regulating the gut microbiota	[51]
(2*E*)-3-[(3*R*)-3,4-dihydro-6,8-dihydroxy-1-oxo-1H-2-benzopyran-3-yl]-2-propenoic-acid (**79**)	*Penicillium* sp. TW58-16	Deep-sea hydrothermal vent sediment, Kueishantao, Taiwan, China	MZ558028	The regulation of gut microbiota contributes to anti-inflammatory effects	[52]
Alternapyrones G (**80**) and H (**81**)	*Arthrinium arundinis* ZSDS-F3	*Phakellia fusca*, Xisha Islands of China	KF693784	Inhibited NO release stronger than 50% at 20 µM	[53]
Alternapyrone D (**82**)	*A. arundinis* ZSDS-F3	*Phakellia fusca*, Xisha Islands of China	KF693784	Inhibited NO release stronger than 50% at 20 µM	[53]
6-alkenylpyrone polyketides alternapyrones E (**83**)	*A. arundinis* ZSDS-F3	The Xisha Islands of China	KF693784	Inhibited NO release stronger than 50% at 20 µM	[53]
Penicilazaphilones F (**84**) and G (**85**)	*Penicillium sclerotiorum* E23Y-1A	Sponge *Holoxea* sp., Quanfu Island, Hainan, China	MW090660	Inhibited NO production in LPS-induced BV-2 cells, IC_50_ = 31.7 ± 1.5 and 34.5 ± 1.4, respectively	[54,55]
Penicilazaphilones I, K, L and N (**86**, **87**, **88** and **89**)	*P. sclerotiorum* E23Y-1A	Sponge, Quanfu Island, Hainan, China	MW090660	Inhibited NO production in LPS-induced RAW 264.7 cells, IC_50_ = 22.63–65.30 µM	[54,55]
Hypocrellone A (**90**)	*P. sclerotiorum* E23Y-1A	Sponge, Quanfu Island, Hainan, China	MW090660	Inhibited NO production in LPS-induced BV-2 cells, IC_50_ = 25.3 ± 2.2 μΜ	[55]
Penicillazaphilone D (**91**)	*P. sclerotiorum* E23Y-1A	Sponge, Quanfu Island, Hainan, China	MW090660	Inhibited NO production in LPS-induced BV-2 cells, IC_50_ = 34.8 ± 1.9 μΜ	[55]
Sclerketide F (**92**)	*Penicillium sclerotiorin* SCNU-F0040	Mangrove *Bruguiera gymnorhiza*, Zhanjiang Mangrove Nature Reserve, Guangdong, China	MW-541637	COX-2 inhibitory activity, IC_50_ = 47.8 μΜ	[56]
8a-epi-hypocrellone A (**93**)	*P. sclerotiorum*	Alga *Grateloupia* sp., Yilan County, Taiwan	KM265451.1	Inhibited the TNF-*α*-induced NF-*κ*B phosphorylation	[57]
8a-epi-eupenicilazaphilone C (**94**)	*P. sclerotiorum*	Alga *Grateloupia* sp., Yilan County, Taiwan	KM265451.1	Promote both TGF-*β*/Smad signaling and transcriptional function	[57]
Hypocrellone A (**95**)	*P. sclerotiorum*	Alga *Grateloupia* sp., Yilan County, Taiwan	KM265451.1	Inhibited the TNF-*α*-induced NF-*κ*B phosphorylation	[57]
Sclerotiorin (**96**)	*P. sclerotiorum*	Alga *Grateloupia* sp., Yilan County, Taiwan	KM265451.1	Inhibited both TGF-*β*/Smad-mediated signaling and transcriptional function	[57]
Isochromophilone IV **(97**)	*P. sclerotiorum*	Alga *Grateloupia* sp., Yilan County, Taiwan	KM265451.1	Inhibited the TNF-*α*-induced NF-*κ*B phosphorylation	[57]
Sclerketide B (**98**) and Sclerketide C (**99**)	*P. sclerotiorum* CHNSCLM-0013	Gorgonian, Weizhou coral reef, South China Sea	KT695601	Inhibited NO production in LPS-induced RAW 264.7 cells, IC_50_ = 3.4 and 17.6 μΜ, respectively	[43]
Isochromophilone IX (**100**)	*P. sclerotiorum* CHNSCLM-0013	Gorgonian, Weizhou coral reef, South China Sea	KT695601	Inhibited NO production in LPS-induced RAW 264.7 cells, IC_50_ = 2.7 μΜ, respectively	[43]
12-Deacetylphomoxanthone A (**101**)	*Diaporthe* sp. SYSU-MS4722	Ascidian, Bay of Da’ao, Guangdong Province, China	OK623372	Inhibited NO production in LPS-induced RAW 264.7 cells, IC_50_ = 6.3 μΜ	[58]
Phomoxanthones A (**102**) and B (**105**)	*Diaporthe* sp. SYSU-MS4722	Ascidian, Bay of Da’ao, Guangdong Province, Chin	OK623372	Inhibited NO production in LPS-induced RAW 264.7 cells, IC_50_ = 7.5 and 8.0 μΜ, respectively	[58]
Dicerandrols B (**103**) and C (**104**)	*Diaporthe* sp. SYSU-MS4722	Ascidian, Bay of Da’ao, Guangdong Province, Chin	OK623372	Inhibited NO production in LPS-induced RAW 264.7 cells, IC_50_ = 6.3 and 7.6 μΜ, respectively	[58]
Deacetylphomoxanthone B (**106**)	*Diaporthe* sp. SYSU-MS4722	Ascidian, Bay of Da’ao, Guangdong Province, Chin	OK623372	Inhibited NO production in LPS-induced RAW 264.7 cells, IC_50_ = 7.8 μΜ	[58]
Epiremisporines G (**107**) and H (**108**)	*Penicillium citrinum* BCRC 09F458	Hazailiao, Dongshi, Chiayi, Taiwan, China	_	Significantly inhibited the production of superoxide ions by fMLP, IC_50_ = 31.68 ± 2.53, and 33.52 ± 0.42 µM, respectively. Positive control ibuprofen, IC_50_ = 28.56 µM	[59]
1,3,5,6-tetrahydroxy-8-methyl-xanthone (**109**)	*Arthrinium arundinis* MA30	Sea anemone, Badouzi	OM761170	Inhibited NO production in LPS-induced BV-2 cells, IC_50_ = 5.3 μΜ	[60]
Arthripenoid C (**110**)	*A. arundinis* MA30	Sea anemone, Badouzi	OM761170	Inhibited NO production in LPS-induced BV-2 cells, IC_50_ = 5.3 μΜ	[60]
Ravenelin (**111**)	*Setosphaeria rostrata*	Mangrove, Prachuap Kiri Khan Province, Thailand	OK047731	Inhibited NO production in LPS-induced RAW 264.7 cells, IC_50_ = 6.27 μΜ Suppressed iNOS and COX-2 expression	[61]
Trichillinoids A-D (**112–115**)	*Trichoderma* sp. G13	Marine fish *Sebastes schlegelii*, Yangma Island, Yantai, China	OQ781262	Inhibited NO production in LPS-induced RAW 264.7 cells, IC_50_ = 14, 14, 16, and 20 µM, respectively	[62]
24-Hydroxybisvertinol (**116**)	*Trichoderma reesei* 4670	Sponge, Shantou, Guangdong Province, China	MH542677	Inhibited NO production in LPS-induced RAW 264.7 cells, IC_50_ = 6.1 μΜ	[63]
Bisvertinol (**117**)	*T. reesei* 4670	Sponge, Shantou, Guangdong Province, China	MH542677	Inhibited NO production in LPS-induced RAW 264.7 cells, IC_50_ = 9.9 μΜ	[63]
Trichobisvertinols A-D (**118-121**)	*T. reesei* 4670	Sponge, Shantou, Guangdong Province, China	MH542677	Inhibited NO production in LPS-induced RAW 264.7 cells, IC_50_ = 5.9, 22 and 24 μΜ, respectively	[63]
12-epi-trichobisvertinol D **(122)**	*T. reesei* 4670	Sponge, Shantou, Guangdong Province, China	MH542677	Inhibited NO production in LPS-induced RAW 264.7 cells, IC_50_ = 32 μΜ	[63]
Trichosorbicillins B (**123**) and C (**124**)	*T. reesei* 4670	Sponge, Shantou, Guangdong Province, China	MH542677	Inhibited NO production in LPS-induced RAW 264.7 cells, IC_50_ = 8.5 and 38 μΜ, respectively	[63]
Trichosorbicillin J (**125**)	*Trichoderma reesei* BGRg-3	Mangrove plant *Avicennia marina*, Guangdong Province, China	OR353740	Potent inhibition of IL-6 45%, and IL-1*β* 21%, respectively, at 25 µM	[64]
Demethylsorbiquinol (**126**)	*T. reesei* BGRg-3	Mangrove plant *Avicennia marina*, Guangdong Province, China	OR353740	Potent inhibition of IL-6 27%, and IL-1*β* 75%, respectively, at 25 µM	[64]
Sorbiquinol (**127**)	*T. reesei* BGRg-3	Mangrove plant *Avicennia marina*, Guangdong Province, China	OR353740	Potent inhibition of IL-6 35%, and IL-1*β*, 58%, respectively, at 25 µM	[64]
13-hydroxy-trichodermolide (**128**)	*T. reesei* BGRg-3	Mangrove plant *Avicennia marina*, Guangdong Province, China	OR353740	47% inhibition of IL-6, 85% inhibition of IL-1*β* at 25 µM	[64]
Trichodimerol (**129**)	*T. reesei* BGRg-3	Mangrove plant *Avicennia marina*, Guangdong Province, China	OR353740	67% inhibition of IL-6, 87% inhibition of IL-1*β* at 25 µM	[64]
Trichopyrone (**130**)	*Penicillium* sp. DM815	Mangrove *Hibiscus tiliaceus* Linnn, Qinglan, Wenchang, Hainan, China	NR_111815.1	Inhibition of LPS-induced iNOS expression in a dose-dependent manner	[36]
Epite-trahydrotrichodimer ether (**131**)	*Penicillium* sp. DM815	Mangrove *Hibiscus tiliaceus* Linnn, Qinglan, Wenchang, Hainan, China	NR_111815.1	Inhibition of LPS-induced iNOS expression in a dose-dependent manner	[39]
(9′R)-tetrahydrotrichodimer ether (**132**)	*Penicillium* sp. DM815	Mangrove *Hibiscus tiliaceus* Linnn, Qinglan, Wenchang, Hainan, China	NR_111815.1	Inhibition of LPS-induced iNOS expression in a dose-dependent manner	[39]
Trichodimerol (**133**)	*Penicillium* sp. DM815	Mangrove *Hibiscus tiliaceus* Linnn, Qinglan, Wenchang, Hainan, China	NR_111815.1	Inhibition of LPS-induced iNOS expression in a dose-dependent manner	[39]
Dihydrotrichodimerol (**134**)	*Penicillium* sp. DM815	Mangrove *Hibiscus tiliaceus* Linnn, Qinglan, Wenchang, Hainan, China	NR_111815.1	Inhibition of LPS-induced iNOS expression in a dose-dependent manner	[39]
Tetrahydrotrichodimerol (**135**)	*Penicillium* sp. DM815	Mangrove *Hibiscus tiliaceus* Linnn, Qinglan, Wenchang, Hainan, China	NR_111815.1	Inhibition of LPS-induced iNOS expression in a dose-dependent manner	[39]
Sorbicillinolides A–G (**136–142**)	*Penicillium rubens* F54	Deep-sea sediment, Pacific Ocean	OR016127	Inhibitory effects on the production of NO and PGE2, inhibition rates of 68.6%, 36.6%, 64.7%, 44.5%, 54.9%, 41.9%, and 44.5%, respectively, at 10 μM	[65]
Sorbicillinolide J (**143**)	*Penicillium rubens* F54	Deep-sea sediment, Pacific Ocean	OR016127	Inhibitory effects on the production of NO and PGE2, inhibition rate of 33.4%, at 10 μM	[65]
Chlorophenol A (**144**)	*Amorosia* sp. SCSIO 4102	Mangrove *Avicennia marina*, Zhanjiang, Guangdong, China	OL826791	Inhibited pro-inflammatory cytokines at the mRNA and protein levels	[36]
α-acetylorcinol (**145**)	*Amorosia* sp. SCSIO 4102	Mangrove *Avicennia marina*, Zhanjiang, Guangdong, China	OL826791	Inhibited pro-inflammatory cytokines at the mRNA and protein levels	[36]
Kojic acid (**146**)	*Amorosia* sp. SCSIO 4102	Mangrove *Avicennia marina*, Zhanjiang, Guangdong, China	OL826791	Inhibited pro-inflammatory cytokines at the mRNA and protein levels	[36]
(+)-Terrein (**147**)	*Aspergillus flavipes* MTCC 5220	Alga *Ulva lactuca*, Goa, India	PRJNA611016	Inhibitory activity against IL-6 and TNF-α, IC_50_ = 8.5 ± 0.68 and 15.76 ± 0.18 µM, respectively	[48]
Sequoiatone B (**148**)	*Penicillium sclerotiorum* CHNSCLM-0013	Gorgonian, Weizhou coral reef, South China Sea	KT695601	Inhibited NO production in LPS-induced RAW 264.7 cells, IC_50_ = 5.2 μΜ	[43]
Diaporspchromanones B–C (**149–150**)	*Diaporthe* sp. XW12-1	Mangrove, Xuwen, Guangdong, China	MW566595.1	IC_50_ (anti-inflammatory) = 19.06 ± 3.60 and 9.56 ± 0.18 μM, respectively, positive control (indomethacin, IC_50_ = 70.33 ± 0.95 μM)	[66]
Phomasparapyrone B (**151**)	*Phomopsis asparagi* LSLYZ-87	Mangrove *Acanthus ilicifolius*, Huizhou Mangrove National Nature Reserve, Guangdong, China	ON341023	Inhibition of LPS-induced NO accumulation on BV-2 cells in a dose-dependent manner	[67]
Pisorhodoptilometrin (**152**)	*Penicillium oxalicum* CLC-MF05	Sponge, Cu Lao Cham islands, Quang Nam, Vietnam	MT597864.1	Inhibited NO production in LPS-induced BV-2 cells, IC_50_ = 15.2 µM	[58]
Guhypoxylonols A (**153**), C (**154**), D (**155**)	*Aspergillus* sp. GXNU-Y45	Mangrove *Acanthus ilicifolius*, Beihai City, China	MT626059	Inhibited NO production in LPS-induced RAW 264.7 cells, IC_50_ = 14.42 ± 0.11, 18.03 ± 0.14, 16.66 ± 0.21, and 21.05 ± 0.13 µM, respectively	[68]
Hypoxylonol B (**156**)	*Aspergillus* sp. GXNU-Y45	Mangrove *Acanthus ilicifolius*, Beihai City, China	MT626059	Inhibited NO production in LPS-induced RAW 264.7 cells, IC_50_ = 21.05 ± 0.13 µM	[68]
Heterocornols T (**157**) and X (**158**)	*Pestalotiopsis heterocornis* XWS03F09	Xisha Islands, China	JN943628.1	Inhibited NOS protein expression in a concentration-dependent manner	[69]
Trypacidin (**159**)	*Talaromyces helicus* SCSIO41311	Cold seep, South China Sea	KT224828	Inhibited NO production in LPS-induced RAW 264.7 cells, IC_50_ = 9.65 μΜ	[70]
Fumiquinone B (**160**)	*T. helicus* SCSIO41311	Cold seep, South China Sea	KT224828	Inhibited NO production in LPS-induced RAW 264.7 cells, IC_50_ = 15.54 μΜ	[70]
Carneusones E-F (**161–162**)	*Aspergillus carneus* GXIMD00543	Sponge, Weizhou islands coral reef, China	OR501447	Inhibited NO production in LPS-induced RAW 264.7 cells, EC_50_ = 34.6 and 20.2 μΜ, respectively	[71]
5-Hydroxy-7-(2′-hydroxypropyl)-2-methyl-chromone (**163**)	*Penicillium oxalicum* CLC-MF05	Sponge, Cu Lao Cham islands, Quang Nam, Vietnam	NR 121232.1	Inhibited NO production in LPS-induced BV-2 cells, IC_50_ = 75.5 μΜ	[72]
Phomtersine A (**164**)	*Phomopsis tersa* FS441	Deep sea in the Indian Ocean	MK592793	Inhibited NO production in LPS-induced RAW 264.7 cells, IC_50_ = 83.57 μΜ	[73]
Steckfusarin A (**165**)	*Penicillium steckii* SCSIO 41040	Green algae *Botryocladia* sp., South China Sea	OP349656	Anti-inflammatory activity at 20 µM	[74]
5-*O*-acetyladenosine (**166**)	*Samsoniella hepiali* W7	Deep-sea sulfide sample, South Atlantic	OR398925	Inhibited NO production in LPS-induced BV-2 cells, inhibition rates of 34.2%, at 1 µM	[46]
Uridine (**167**)	*S. hepiali* W7	Deep-sea sulfide sample, South Atlantic	OR398925	Inhibited NO production in LPS-induced BV-2 cells, inhibition rates of 30.7%, at 1 µM	[46]
Sclerotioloid B (**168**)	*Aspergillus sclerotiorum* ST0501	Guangdong, China	MT534582	Inhibited NO production in LPS-induced RAW 264.7 cells, inhibition rate of 28.92%, postive control dexamethasone (25.87%)	[75]
Sclerotiamide J (**169**)	*Aspergillus sclerotiorum* LZDX-33-4	Gorgonian, South China Sea	OK012383.1	Inhibitory effect on the expression of LDH and IL-1*β* in BV-2 cells	[76]
Sclerotiamides K (**170**)	*A. sclerotiorum* LZDX-33-4	Gorgonian, South China Sea	OK012383.1	Inhibitory effect on the expression of LDH and IL-1*β* in BV-2 cells	[76]
Sclerotiamides O-Q (**171–173**)	*A. sclerotiorum* LZDX-33-4	Gorgonian, South China Sea	OK012383.1	Inhibitory effect on the expression of LDH and IL-1*β* in BV-2 cells	[76]
Notamide X (**174**)	*A. sclerotiorum* LZDX-33-4	Gorgonian, South China Sea	OK012383.1	Inhibitory effect on the expression of LDH and IL-1*β* in BV-2 cells	[76]
Notamide Z (**175**)	*A. sclerotiorum* LZDX-33-4	Gorgonian, South China Sea	OK012383.1	Inhibitory effect on the expression of LDH and IL-1*β* in BV-2 cells	[76]
Notamide R (**176**)	*A. sclerotiorum* LZDX-33-4	Gorgonian, South China Sea	OK012383.1	Inhibitory effect on the expression of LDH and IL-1*β* in BV-2 cells	[76]
(−)-notamide A (**177**)	*A. sclerotiorum* LZDX-33-4	Gorgonian, South China Sea Gorgonian, South China Sea	OK012383.1	Inhibitory effect on the expression of LDH and IL-1*β* in BV-2 cells	[76]
Notamide I (**178**)					
Notamide F (**179**)	*A. sclerotiorum* LZDX-33-4	Gorgonian, South China Sea	OK012383.1	Inhibitory effect on the expression of LDH and IL-1*β* in BV-2 cells	[76]
Sclerotiamide (**180**)	*A. sclerotiorum* LZDX-33-4	Gorgonian, South China Sea	OK012383.1	Inhibitory effect on the expression of LDH and IL-1*β* in BV-2 cells	[76]
Sclerotiamide B (**181**)	*A. sclerotiorum* LZDX-33-4	Gorgonian, South China Sea	OK012383.1	Inhibitory effect on the expression of LDH and IL-1*β* in BV-2 cells	[76]
Equisetin (**182**)	*Fusarium equiseti*	Sponge, Xuwen County, Zhanjiang, China	SCSIO 41019	EQST inhibits macrophage inflammatory response in vitro	[77]
Penpaxilloids A (**183**), C (**184**), D (**185**)	*Penicillium* sp. ZYX-Z-143	Arthropod *Dardanus scutellatus*, Yinyu Island, Hainan	ON386189	Inhibited NO production in LPS-induced RAW 264.7 cells, IC_50_ = 7.11, 27.25 and 33.09 μΜ, respectively	[78]
7-methoxypaxilline-13-ene (**186**)	*Penicillium* sp. ZYX-Z-143	Arthropod *Dardanus scutellatus*, Yinyu Island, Hainan	ON386189	Inhibited NO production in LPS-induced RAW 264.7 cells, IC_50_ = 38.79 μΜ	[78]
Schipenindolene A (**187**)	*Penicillium* sp. ZYX-Z-143	Arthropod *Dardanus scutellatus*, Yinyu Island, Hainan	ON386189	Inhibited NO production in LPS-induced RAW 264.7 cells, IC_50_ = 11.87 μΜ	[78]
21-isopentenylpaxilline (**188**)	*Penicillium* sp. ZYX-Z-143	Arthropod *Dardanus scutellatus*, Yinyu Island, Hainan	ON386189	Inhibited NO production in LPS-induced RAW 264.7 cells, IC_50_ = 32.95 μΜ	[78]
Penerpene E (**189**)	*Penicillium* sp. ZYX-Z-143	Arthropod *Dardanus scutellatus*, Yinyu Island, Hainan	ON386189	Inhibited NO production in LPS-induced RAW 264.7 cells, IC_50_ = 23.89 μΜ	[78]
Paspalinine (**190**)	*Penicillium* sp. ZYX-Z-143	Arthropod *Dardanus scutellatus*, Yinyu Island, Hainan	ON386189	Inhibited NO production in LPS-induced RAW 264.7 cells, IC_50_ = 19.34 μΜ	[78]
4a-demethylpaspaline-4a-carboxylic acid (**191**)	*Penicillium* sp. ZYX-Z-143	Arthropod *Dardanus scutellatus*, Yinyu Island, Hainan	ON386189	Inhibited NO production in LPS-induced RAW 264.7 cells, IC_50_ = 28.22 μΜ	[78]
Paxilline D (**192**)	*Penicillium* sp. ZYX-Z-143	Arthropod *Dardanus scutellatus*, Yinyu Island, Hainan	ON386189	Inhibited NO production in LPS-induced RAW 264.7 cells, IC_50_ = 4.46 μΜ	[78]
		Arthropod *Dardanus scutellatus*, Yinyu Island in South China’s Hainan province			
7-methoxypaxilline (**193**)	*Penicillium* sp. ZYX-Z-143	Arthropod *Dardanus scutellatus*, Yinyu Island, Hainan	ON386189	Inhibited NO production in LPS-induced RAW 264.7 cells, IC_50_ = 22.88 μΜ	[78]
Terreusinones B (**194**) and C (**195**)	*Aspergillus tamarii* MCCF102	Sponge, Vizhinjam, Southwest coast of India	JAGJCD000000000	Inhibited NO production in LPS-induced RAW 264.7 cells, IC_50_ = 0.032, 0.046 and 0.096μΜ. respectively	[79]
Terreusinone (**196**)	*A. tamarii* MCCF102	Sponge, Vizhinjam, Southwest coast of India	JAGJCD000000000	Inhibited NO production in LPS-induced RAW 264.7 cells, IC_50_ = 0.032 μΜ	[79]
Chaetominine (**197**)	*Talaromyces helicus* SCSIO41311	Cold seep, South China Sea	KT224828	Inhibited NO production in LPS-induced RAW 264.7 cells, IC_50_ = 103.2 μΜ	[70]
Isotryptoquivaline F (**198**)	*T. Helicus* SCSIO41311	Cold seep, South China Sea	KT224828	Inhibited NO production in LPS-induced RAW 264.7 cells, IC_50_ = 26.51 μΜ	[70]
Fumiquinazoline F (**199**)	*T. Helicus* SCSIO41311	Cold seep, South China Sea	KT224828	Inhibited NO production in LPS-induced RAW 264.7 cells, IC_50_ = 21.35 μΜ	[70]
12,13-dihydroxyfumitremorgin C (**200**)	*T. Helicus* SCSIO41311	Cold seep, South China Sea	KT224828	Inhibited NO production in LPS-induced RAW 264.7 cells, IC_50_ = 24.95 μΜ	[70]
**C**yclotryprostatin B (**201**)	*T. Helicus* SCSIO41311	Cold seep, South China Sea	KT224828	Inhibited NO production in LPS-induced RAW 264.7 cells, IC_50_ = 29.58 μΜ	[70]
Azaspirofurans A (**202**)	*T. Helicus* SCSIO41311	Cold seep, South China Sea	KT224828	Inhibited NO production in LPS-induced RAW 264.7 cells, IC_50_ = 9.65 μΜ	[70]
14-norpseurotin A (**203**)	*T. Helicus* SCSIO41311	Cold seep, South China Sea Cold seep, South China Sea	KT224828	Inhibited NO production in LPS-induced RAW 264.7 cells, IC_50_ = 32.37 μΜ	[70]
11-O methylpseurotin A (**204**)	*T. Helicus* SCSIO41311	Cold seep, South China Sea	KT224828	Inhibited NO production in LPS-induced RAW 264.7 cells, IC_50_ = 32.22 μΜ	[70]
Fumigaclavine C (**205**)	*T. Helicus* SCSIO41311	Cold seep, South China Sea	KT224828	Inhibited NO production in LPS-induced RAW 264.7 cells, IC_50_ = 23.46 μΜ	[70]
Oxaline (**206**)	*Penicillium oxalicum* CLC-MF05	Sponge, Cu Lao Cham islands, Quang Nam, Vietnam	MT597864.1	Inhibited NO production in LPS-induced BV-2 cells, IC_50_ = 9.2 μΜ	[72]
Variotin B (**207**)	*Aspergillus unguis* IV17-109	Deep sea, Indian Ocean	OL700797	Inhibited NO production in LPS-induced RAW 264.7 cells, IC_50_ = 20.0 μΜ	[80]
Benzomalvin E (**208**)	*Metarhizium* sp. P2100	Seawater, Qingdao Huiquan Bay, Yellow Sea	OP028052	Inhibited NO production in LPS-induced RAW 264.7 cells, IC_50_ = 37.08 μΜ	[81]
Methylviridicatin (**209**)	*Metarhizium* sp. P2100	Seawater, Qingdao Huiquan Bay, Yellow Sea	OP028052	Inhibited NO production in LPS-induced RAW 264.7 cells, IC_50_ = 37.48 μΜ	[81]
Isoechinulin B (**210**)	*Aspergillus* sp. nFS445	Deep sea, Indian Ocean	MW386823	Inhibited NO production in LPS-induced RAW 264.7 cells, IC_50_ = 16 ± 1.3 µM, positive control aminoguanidine (IC_50_, 23.7 µM)	[82]
Aspechinulins B (**211**) and C (**213**)	*Aspergillus* sp. nFS445	Sponge, Indian Ocean	MW386823	Inhibited NO production in LPS-induced RAW 264.7 cells, IC_50_ = 20 ± 0.28 and 25 ± 1.7 µM, respectively	[82]
Neoechinulin B (**212**)	*Aspergillus* sp. nFS445	Sponge, Indian Ocean	MW386823	Inhibited NO production in LPS-induced RAW 264.7 cells, IC_50_ = 89 ± 2.0 µM	[82]
Cryptoechinuline G (**214**)	*Aspergillus* sp. nFS445	Sponge, Indian Ocean	MW386823	Inhibited NO production in LPS-induced RAW 264.7 cells, IC_50_ = 37 ± 0.75 µM	[82]
Isoechinulin A (**215**)	*Aspergillus* sp. nFS445	Sponge, Indian Ocean	MW386823	Inhibited NO production in LPS-induced RAW 264.7 cells, IC_50_ = 57 ± 2.3 µM	[82]
(−)-cyclopenol (**216**)	*Aspergillus austroafricanus* Y32-2	Indian Ocean	MK267449	Inhibited NO production in LPS-induced RAW 264.7 cells at 70 µg/mL	[83]
Cyclopenin (**217**)	*A. austroafricanus* Y32-2	Indian Ocean	MK267449	Inhibited NO production in LPS-induced RAW 264.7 cells at 130 µg/mL	[83]
Virdicatol (**218**)	*austroafricanus* Y32-2	Indian Ocean	MK267449	Inhibited NO production in LPS-induced RAW 264.7 cells at 30 µg/mL	[83]
Phomtersine A (**219)**	*Phomopsis tersa* FS441	Deep sea, Indian Ocean	MK592793	Inhibited NO production in LPS-induced RAW 264.7 cells, IC_50_ = 83.57 μΜ	[84]
Penifuranone A (**220**)	*Penicillium crustosum* SCNU-F0006	Mangrove, Yangjiang Hailing Island Mangrove Wetland Park, China	MH345907	Inhibited NO production in LPS-induced RAW 264.7 cells, IC_50_ = 42.2 μΜ	[85]
(±)-penicamide A [(−)-**221** and (+)-**222**]	*Penicillium* sp. 4829	*Styela plicata*, Bay of Da’ao, Guangdong, China	MH465534	Inhibited NO production in LPS-induced RAW 264.7 cells, IC_50_ = 27.2 and 47.5 μΜ, respectively	[86]
Penicamide B (**223**)	*Penicillium* sp. 4829	*Styela plicata*, Bay of Da’ao, Guangdong, China	MH465534	Inhibited NO production in LPS-induced RAW 264.7 cells, IC_50_ = 45.9 ± 2.0 μΜ	[86]
(S)-2-(2-hydroxypropanamido) benzamide (**224**)	*Penicillium* sp. 4829	*Styela plicata*, Bay of Da’ao, Guangdong, China	MH465534	Inhibited NO production in LPS-induced RAW 264.7 cells, IC_50_ = 21.8 ± 1.3 µM, respectively	[86]
Penipiperazine A (**225**)	*Penicillium brasilianum* HBU-136	Bohai Sea, China	MH377073	Inhibited the expression of inflammatory factors at 25.0 µM	[87]
Metabolite (**226**)	*P. brasilianum* HBU-136	Bohai Sea, China	MH377073	Inhibited the expression of inflammatory factors at 25.0 µM	[87]
Cytochalasins Z24 (**227**)	*Eutypella scoparia* GZU-4-19Y	Xuwen, Guangdong province, China	OM920979	Inhibited NO production in LPS-induced RAW 264.7 cells, IC_50_ = 17.1 μΜ	[30]
Aspercerebroside A (**228**)	*Aspergillus* sp.	Dongshan Island, Fujian Province	2167894	Inhibited NO production in LPS-induced RAW 264.7 cells, at 30 and 40 μg/mL	[88]
Hortacerebrosides A (**229**) and B (**230**)	*Hortaea werneckii*	Sponge, Danzhou, Hainan, China	HN-YPG-2-5	Inhibited NO production in LPS-induced RAW 264.7 cells, IC_50_ = 7 and 5 μΜ, respectively	[89]
Acrepeptins A (**231**) and C (**232**)	*Acremonium* sp. NTU492	Red alga *Mastophora rosea*, Taiwan, China	KY753131	Inhibited NO production in LPS-induced BV-2 cells, IC_50_ = 12.0 and 10.6 μΜ, respectively	[90]
Pseudoviridinutans A−G (**233−239**)	*Aspergillus pseudoviridinutans* TW58-5	Ahydrothermal vent sediment, Kueishantao, Taiwan, China	OQ405296	Inhibited NO production in LPS-induced RAW 264.7 cells	[91]
Sterolester (**240**)	*Penicillium oxalicum* HL-44	Soft coral *Sinularia gaweli*, Xisha, South China Sea	MG585101.1	Strong anti-inflammatory activity at 20 μΜ	[92]
22-Tetraen-3-one (**241**)	*P. oxalicum* HL-44	Soft coral *Sinularia gaweli*, Xisha, South China Sea	MG585101.1	Strong anti-inflammatory activity at 20 μΜ	[92]
Ganodermaside (**242**)	*P. oxalicum* HL-44	Soft coral *Sinularia gaweli*, Xisha, South China Sea	MG585101.1	Strong anti-inflammatory activity at 20 μΜ	[92]
22-Tetraen-3-one (**243**)	*P. oxalicum* HL-44	Soft coral *Sinularia gaweli*, Xisha, South China Sea	MG585101.1	Strong anti-inflammatory activity at 20 μΜ	[92]
Isocyathisterol (**244**)	*P. oxalicum* HL-44	Soft coral *Sinularia gaweli*, Xisha, South China Sea	MG585101.1	Strong anti-inflammatory activity at 20 μΜ	[92]
Herbarulide (**245**)	*P. oxalicum* HL-44	Soft coral *Sinularia gaweli*, Xisha, South China Sea	MG585101.1	Strong anti-inflammatory activity at 20 μΜ	[92]
Dankasterone A (**246**)	*P. oxalicum* HL-44	Soft coral *Sinularia gaweli*, Xisha, South China Sea	MG585101.1	Strong anti-inflammatory activity at 20 μΜ	[92]
(22*E*,24*R*)-ergosta-7,22-dien-3*β*,5*α*-diol-6-one (**247**)	*P. oxalicum* HL-44	Soft coral *Sinularia gaweli*, Xisha, South China Sea	MG585101.1	Strong anti-inflammatory activity at 20 μΜ	[92]
(22*E*,24*R*)-ergosta-7,22-dien-3*β*,5*α*,9*α*-trihydroxy-6-one (**248**)	*P. oxalicum* HL-44	Soft coral *Sinularia gaweli*, Xisha, South China Sea	MG585101.1	Strong anti-inflammatory activity at 20 μΜ	[92]
(22*E*,24*R*)-3*β*-hydroxyergosta-5,8,22-trien-7-one (**249**)	*P. oxalicum* HL-44	Soft coral *Sinularia gaweli*, Xisha, South China Sea	MG585101.1	Strong anti-inflammatory activity at 20 μΜ	[92]
22-triene-3*β*-ol (**250**)	*P. oxalicum* HL-44	Soft coral *Sinularia gaweli*, Xisha, South China Sea	MG585101.1	Strong anti-inflammatory activity at 20 μΜ	[92]
(22*E*,24*R*)-7*α*-methoxy-5*α*,6*α*-epoxyergosta-8(14),22-dien-3*β*-ol (**251**)	*P. oxalicum* HL-44	Soft coral *Sinularia gaweli*, Xisha, South China Sea	MG585101.1	Strong anti-inflammatory activity at 20 μΜ	[92]
(22E,24R)-6-acetoxy-ergosta-7,22-dien-3β,5α,6β-triol (**252**)	*P. oxalicum* HL-44	Soft coral *Sinularia gaweli*, Xisha, South China Sea	MG585101.1	Strong anti-inflammatory activity at 20 μΜ	[92]
(22*E*,24*R*)-5*α*,8*α*-epidioxyergosta-6,9(11),22-trien-3*β*-ol (**253**)	*P. oxalicum* HL-44	Soft coral *Sinularia gaweli*, Xisha, South China Sea	MG585101.1	Strong anti-inflammatory activity at 20 μΜ	[92]
(22*E*,24*R*)-5*α*,8*α*-epidioxyergosta-6,22-dien-3*β*-ol (**254**)	*P. oxalicum* HL-44	Soft coral *Sinularia gaweli*, Xisha, South China Sea	MG585101.1	Strong anti-inflammatory activity at 20 μΜ	[92]
Demethylincisterol A3 (**255**)	*P. oxalicum* HL-44	Soft coral *Sinularia gaweli*, Xisha, South China Sea	MG585101.1	Strong anti-inflammatory activity at 20 μΜ	[92]
Ergosterdiacids A and B (**256** and **257**)	*Aspergillus* sp.	Mangrove *Aegiceras corniculatum*, Thailand	2167894	Inhibited NO production in LPS-induced RAW 264.7 cells, IC_50_ = 4.5 and 3.6 μΜ, respectively	[93]
(22*E*,24*R*)-ergosta-5,7,22-trien-3*β-*ol (**258**)	*Amorosia* sp. SCSIO 4102	Mangrove *Avicennia marina*, Zhanjiang, Guangdong, China	OL826791	Inhibited excessive LPS-induced production of NO and pro-inflammatory cytokines at the mRNA and protein levels	[36]
Ergosterol (**259**)	*Samsoniella hepiali* W7	Deep-sea sulfide sample, South Atlantic	NR_160318.1	Inhibited NO production in LPS-induced BV-2 cells, inhibition rate of 32.9% (1 μΜ)	[46]
4-hydroxy-3-(3-methylbut-2-en-1-yl)-benzaldehyde (**260**)	*Aspergillus terreus* C23-3	Coral, Xuwen natural reserve located, South China Sea	_	Inhibited the MAPK signaling pathway in RAW264.7 cells	[94]
(S)-3-(2,3-dihydroxy-3-methylbutyl)-4-hydroxybenzalde-hydehave (**261**)	*Aspergillus terreus* C23-3	Coral, Xuwen natural reserve located, South China Sea	_	Inhibited the MAPK signaling pathway in RAW264.7 cells	[94]
Alternate C (**262**)	*Pleosporales* sp. SF-7343	King George Island, Antarctica	MK785420	Inhibition of IL-6 and IL-8	[47]
Talarocyclopentas A (**263**), B (**264**) and C (**265**)	*Talaromyces assiutensis* JTY2	Mangrove *Ceriops tagal*, South China Sea	JN899320.1	Inhibited NO production in LPS-induced RAW 264.7 cells, IC_50_ = 3.38, 6.26, and 12.56 μΜ, respectively	[95]
Asperitaconic B (**266**)	*T. assiutensis* JTY2	Mangrove *Ceriops tagal*, South China Sea	JN899320.1	Inhibited NO production in LPS-induced RAW 264.7 cells, IC_50_ = 15.63 μΜ	[95]
5-[(3*E*,5*E*)-3,5-nonadienyl]-1,3-benzenediol (**267**)	*Aspergillus* sp.	Brown alga *Saccharina cichorioides*, South China Sea	2167894	Inhibited NO production in LPS-induced RAW 264.7 cells, IC_50_ = 6.0 μΜ	[96]
3,7-dihydroxy-1,9-dimethyldibenzofuran (**268)**	*Aspergillus sydowii* MCCC 3A00324	Deep sea sediment, South Atlantic Ocean	MN918102	Inhibited NO production in LPS-induced BV-2 cells, 94.4% (10 μΜ)	[24]
Chlorophenol A (**269**)	*Amorosia* sp. SCSIO 4102	Mangrove *Avicennia marina*, Zhanjiang, Guangdong, China	OL826791	Inhibited excessive LPS-induced production of NO and pro-inflammatory cytokines at the mRNA and protein levels	[36]
*α*-acetylorcinol (**270**)	*Amorosia* sp. SCSIO 4102	Mangrove *Avicennia marina*, Zhanjiang, Guangdong, China	OL826791	Inhibited excessive LPS-induced production of NO and pro-inflammatory cytokines at the mRNA and protein levels	[36]
Monod-ictyphenone (**271**)	*Diaporthe* sp. SYSU-MS4722	Shenzhen City, Guangdong, Province, China	OK623372	Inhibited NO production in LPS-induced RAW 264.7 cells, IC_50_ = 40.8 μΜ	[38]
12-hydroxysorbicillin (**272**)	*Trichoderma reesei* 4670	Sponge, Shantou, Guangdong Province, China	MH542677	Inhibited NO production in LPS-induced RAW 264.7 cells, IC_50_ = 6.8 μΜ	[63]
8,9-Dihydro-12-hydroxysorbicillin (**273**)	*T. reesei* 4670	Sponge, Shantou, Guangdong Province, China	MH542677	Inhibited NO production in LPS-induced RAW 264.7 cells, IC_50_ = 2.9 μΜ	[63]
Trichosorbicillin E (**274**)	*T. reesei* 4670	Sponge, Shantou, Guangdong Province, China	MH542677	Inhibited NO production in LPS-induced RAW 264.7 cells, IC_50_ = 0.94 μΜ	[63]
Trichosorbicillin F (**275**)	*T. reesei* 4670	Sponge, Shantou, Guangdong Province, China	MH542677	Inhibited NO production in LPS-induced RAW 264.7 cells, IC_50_ = 6.1 μΜ	[63]
Isotrichosorbicillin E (**276**)	*T. reesei* 4670	Sponge, Shantou, Guangdong Province, China	MH542677	Inhibited NO production in LPS-induced RAW 264.7 cells, IC_50_ = 12 μΜ	[63]
Sohirnone A (**277**)	*T. reesei* 4670	Sponge, Shantou, Guangdong Province, China	MH542677	Inhibited NO production in LPS-induced RAW 264.7 cells, IC_50_ = 14 μΜ	[63]
2′,3′-dihydrosorbicillin (**278**)	*T. reesei* 4670	Sponge, Shantou, Guangdong Province, China	MH542677	Inhibited NO production in LPS-induced RAW 264.7 cells, IC_50_ = 13 μΜ	[63]
(2E,4E)-1-(2,6-dihydroxy-3,5-dimethylphenyl) hexa-2,4-dien-1-one (**279**)	*T. reesei* 4670	Sponge, Shantou, Guangdong Province, China	MH542677	Inhibited NO production in LPS-induced RAW 264.7 cells, IC_50_ = 3.3 μΜ	[63]
Trichosorbicillin I (**280**)	*T. reesei* 4670	Sponge, Shantou, Guangdong Province, China	MH542677	Inhibited NO production in LPS-induced RAW 264.7 cells, IC_50_ = 13 μΜ	[63]
4-carboxy-5-((1*Z*,3*E*)-1,3-heptadien-1-yl)-1,3-benzenediol (**281**)	*Penicillium* sp. TW58-16	Deep-sea hydrothermal vent sediment, Kueishantao, Taiwan, China	MZ558028	Regulation of gut microbiota contributes to anti-inflammatory effects	[52]
5-((1*Z*,3*E*)-4-carboxy-1,3-butadienyl-1-yl)-1,3-benzenediol (**282**)	*Penicillium* sp. TW58-16	Deep-sea hydrothermal vent sediment, Kueishantao, Taiwan, China	MZ558028	Regulation of gut microbiota contributes to anti-inflammatory effects	[52]
3,4-dihydroxybenzeneaceticacid (**283**)	*Penicillium* sp. TW58-16	Deep-sea hydrothermal vent sediment, Kueishantao, Taiwan, China	MZ558028	Regulation of gut microbiota contributes to anti-inflammatory effects	[52]
Scordyol C (**284**)	*Aspergillus carneus* GXIMD00543	Sponge, Weizhou islands coral reef, China	OR501447	Inhibited NO production in LPS-induced RAW 264.7 cells, IC_50_ = 26.8 ± 1.7μΜ	[72]
3,7-dihydroxy-1,9-dimethyldibenzofuran (**285**)	*Aspergillus carneus* GXIMD00543	Sponge, Weizhou islands coral reef, China	OR501447	Inhibited NO production in LPS-induced RAW 264.7 cells, IC_50_ = 2.9 ± 0.1 μΜ	[72]
